# Ectopic expression of the transcription factor ONECUT3 drives a complex karyotype in myelodysplastic syndromes

**DOI:** 10.1172/JCI172468

**Published:** 2024-02-22

**Authors:** Yingwan Luo, Xiaomin Feng, Wei Lang, Weihong Xu, Wei Wang, Chen Mei, Li Ye, Shuanghong Zhu, Lu Wang, Xinping Zhou, Huimin Zeng, Liya Ma, Yanling Ren, Jie Jin, Rongzhen Xu, Gang Huang, Hongyan Tong

**Affiliations:** 1 Department of Hematology, The First Affiliated Hospital, Zhejiang University School of Medicine, Hangzhou, Zhejiang, China.; 2 Department of Cell Systems and Anatomy, Department of Pathology and Laboratory Medicine, UT Health San Antonio, Joe R. and Teresa Lozano Long School of Medicine, Mays Cancer Center at UT Health San Antonio, San Antonio, Texas, USA.; 3 Stanford Genome Technology Center, Palo Alto, California, USA.; 4 Greater Bay Area Institute of Precision Medicine (Guangzhou), Fudan University, Nansha District, Guangzhou, China.; 5 Department of Pediatrics, Peking University People’s Hospital, Beijing, China.; 6 Department of Hematology, The Second Affiliated Hospital, School of Medicine,; 7 Cancer Center, and; 8 Zhejiang Provincial Key Lab of Hematopoietic Malignancy, Zhejiang University, Hangzhou, Zhejiang, China.

**Keywords:** Hematology, Bone marrow, Genetic instability

## Abstract

Chromosomal instability is a prominent biological feature of myelodysplastic syndromes (MDS), with over 50% of patients with MDS harboring chromosomal abnormalities or a complex karyotype (CK). Despite this observation, the mechanisms underlying mitotic and chromosomal defects in MDS remain elusive. In this study, we identified ectopic expression of the transcription factor ONECUT3, which is associated with CKs and poorer survival outcomes in MDS. ONECUT3-overexpressing cell models exhibited enrichment of several notable pathways, including signatures of sister chromosome exchange separation and mitotic nuclear division with the upregulation of *INCENP* and *CDCA8* genes. Notably, dysregulation of chromosome passenger complex (CPC) accumulation, besides the cell equator and midbody, during mitotic phases consequently caused cytokinesis failure and defective chromosome segregation. Mechanistically, the homeobox (HOX) domain of ONECUT3, serving as the DNA binding domain, occupied the unique genomic regions of *INCENP* and *CDCA8* and transcriptionally activated these 2 genes. We identified a lead compound, C5484617, that functionally targeted the HOX domain of ONECUT3, inhibiting its transcriptional activity on downstream genes, and synergistically resensitized MDS cells to hypomethylating agents. This study revealed that ONECUT3 promoted chromosomal instability by transcriptional activation of *INCENP* and *CDCA8*, suggesting potential prognostic and therapeutic roles for targeting high-risk MDS patients with a CK.

## Abstract 2

Ectopic expression of the transcription factor ONECUT3 is a driver of chromosomal abnormalies in myelodysplastic syndromes.

## Graphical abstract

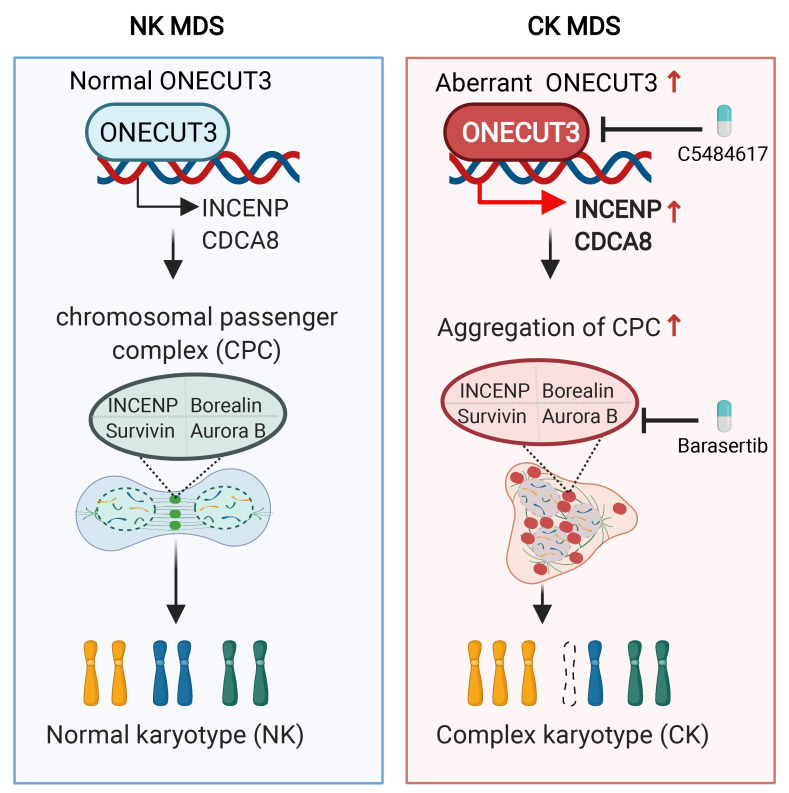

## Introduction

Myelodysplastic syndromes (MDS) refer to a group of clonal hematopoietic disorders characterized by ineffective hematopoiesis and morphological dysplasia (1). Recent studies indicate that up to 14%–25% of patients with de novo MDS harbor a complex karyotype (CK) during initial cytogenetic evaluation (2–4). CK, which refers to 3 or more independent chromosomal abnormalities identified by several complementary techniques, is well established as a poor risk factor for patients with MDS, resulting in a poor prognosis and progression of treatment-related secondary MDS (t-MDS) (5, 6). In recent years, most studies have focused on the associations of TP53 mutation or deactivation with CK (7–9). Hitherto, besides TP53, few genes were reported to be associated with cytogenetic aberration in MDS (10, 11).

ONECUT3 belongs to the ONECUT family, an ancient homeobox transcription factor (TF) superfamily (12). To date, research on ONECUT3 has focused on the physiological regulation of embryo development, while the roles of ONECUT3 in cancer remain poorly understood (13–15). From our consolidated clinical data, high levels of ONECUT3 are positively associated with CK incidences in WT TP53 MDS. Given that a single TF can modulate multiple downstream genes, ONECUT3, a potential master TF of MDS with CK, could be targeted for more effective MDS therapy, especially aggressive MDS.

To fill this knowledge gap, we conducted RNA-Seq to find the CK-associated TF, which showed that ONECUT3 was upregulated in MDS with CK, independent of *TP53* mutation. Intriguingly, ONECUT3 predicted poor survival of patients with MDS. Overexpression (OE) of ONECUT3 induced the enrichment of the mitosis process–related genes *INCENP* and *CDCA8*, as ONECUT3 bound to genomic regions of *INCENP* and *CDCA8*, driving mitotic defects and leading to multinucleation and an aberrant karyotype. Importantly, high ONECUT3 levels were associated with poor sensitivity to chemotherapy, suggesting that ONECUT3-targeted therapy may benefit the chemosensitization of MDS patients with CK. Our study provided the evidence of ONECUT3 as a TF and regulator in MDS with CK, revealing ONECUT3 as a potential therapeutic target for patients with MDS.

## Results

### High ONECUT3 expression is correlated with CK in MDS.

**Figure 1:**
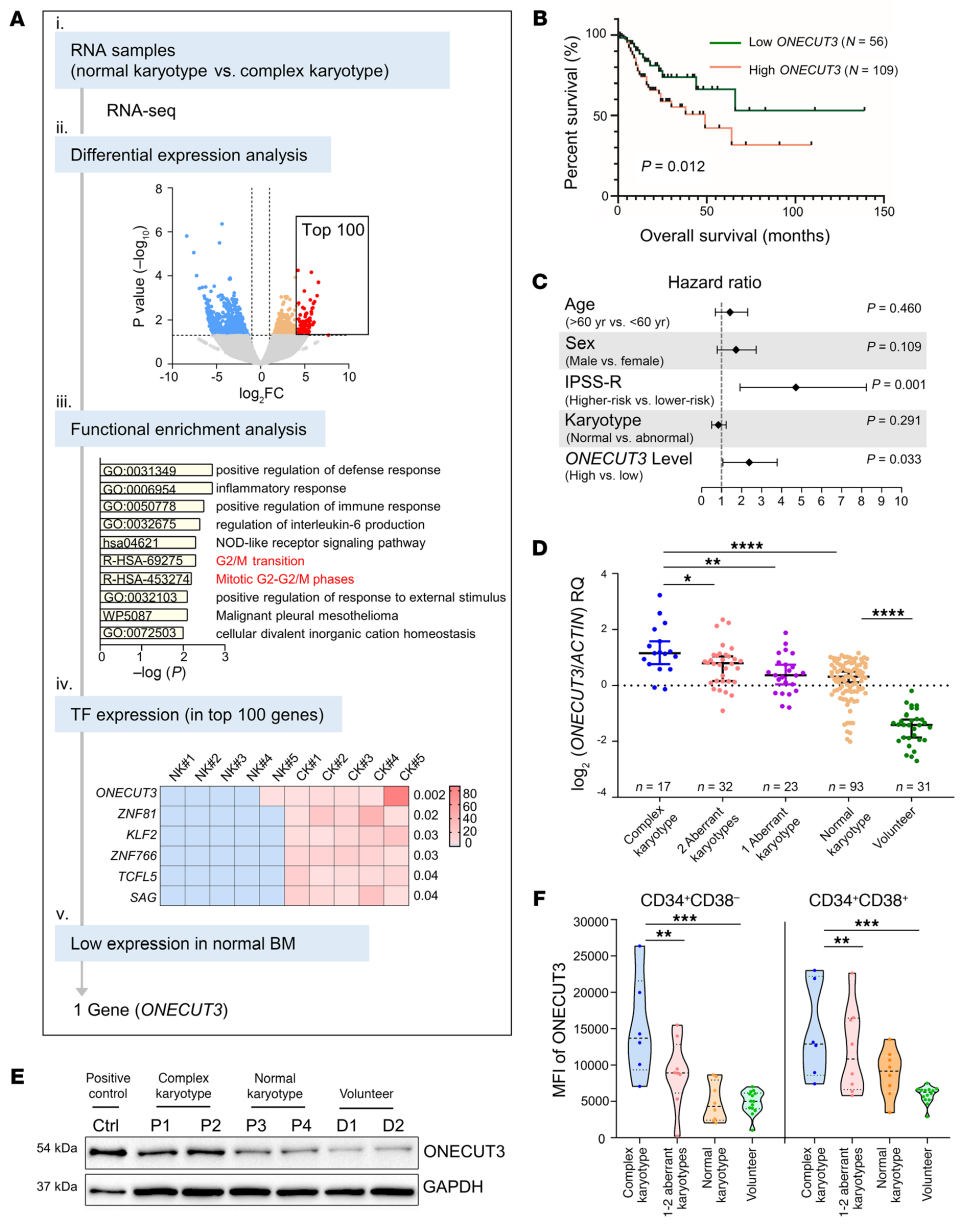
A high level of the TF ONECUT3 is correlated with a CK in MDS. (**A**) Schematic illustration of paired RNA-Seq to find the potential transcription regulator for a CK. (i) RNA-Seq was performed on RNA samples from 5 paired patients with MDS with a CK or a NK. Each sample was matched for sex, age, and BM blasts (Supplemental Table 1). (ii) Volcano map showed the differential expression genes. Blue dots represent downregulated genes in the CK versus the NK (*P* < 0.05); orange dots represent upregulated genes (*P* < 0.05, log_2_ fold change [FC] <4.7 in CK); and red dots indicate the top 100 upregulated genes (*P* < 0.05, log_2_ FC >4.7) in Supplemental Table 2. (iii) Functional enrichment analysis of the top 100 upregulated genes by Metascape (metascape.org). (iv) Heatmap visualization of the mRNA abundance (normalized to fragments per kilobase per million mapped reads [FPKM]) for 6 TFs among the top 100 genes. (v) Only *ONECUT3* was expressed at relatively low levels in the BM samples (see Supplemental Figure 1, A and B). (**B**–**D**) Quantitative real-time PCR was performed to measure mRNA levels of *ONECUT3* in 165 MDS patients harboring WT TP53 with different cytogenetic aberrations and 31 volunteers. The mean value ± 1 SD for the healthy donor was used as a cutoff. (**B**) Kaplan-Meier survival curves showing the OS of patients with low or high expression of *ONECUT3*. (**C**) The forest plot was generated based on the multivariable analysis of factors associated with OS. (**D**) Each data point represents *ONECUT3* expression for an individual patient. RQ, relative quantification. (**E**) Western blot analysis was applied to detect endogenous ONECUT3 expression using a homemade antibody (Supplemental Figure 1, C and D). A positive control (Ctrl) was achieved by transfecting HEK293T cells with pcDNA3.1-hONECUT3 for 48 hours. The lysate was from the BM mononuclear cells of MDS patients with a CK (P1 and P2), a NK (P3 and P4), and 2 volunteer donors of HSPCs (D1 and D2). (**F**) ONECUT3 MFI detected by flow cytometry (see gating strategy in Supplemental Figure 1E) in CD34^+^CD38^–^ (left panel) and CD34^+^CD38^+^ (right panel) of BM mononuclear cells from volunteer donors of HSPCs (*n* = 14), MDS patients with a NK (*n* = 8), and MDS patients with an aberrant karyotype (1–2 cytogenetic aberrations, *n* = 8; CK, *n* = 6). Statistical analysis was performed using 1-way ANOVA with Tukey’s multiple-comparison test (**D** and **F**). *P < 0.05, **P < 0.01, and ***P < 0.001, and ****P < 0.0001.

To identify potential master transcriptional regulators associated with CK in MDS, we performed differential analysis of RNA-Seq (CK vs. normal karyotype [NK]). Of note, these patients were WT for *TP53* (Supplemental Table 1; supplemental material available online with this article; https://doi.org/10.1172/JCI172468DS1). TFs with highly aberrant expression were screened from the top 100 upregulated genes (Figure 1A and Supplemental Table 2), and *ONECUT3* was found to be expressed at low levels in normal bone marrow (BM) cells (Figure 1A and Supplemental Figure 1, A and B). High *ONECUT3* expression levels were identified as a predictor of poor overall survival (OS) in univariable and multivariable analyses (Figure 1, B and C, and Supplemental Table 3).

We subsequently expanded the sample size to address the relationship between *ONECUT3* expression and a CK (*n* = 165). mRNA levels of *ONECUT3* were correlated with chromosomal karyotype grouping (*P* = 0.009) (Supplemental Table 4). *ONECUT3* abundance gradually increased in the following groups: healthy volunteers, MDS patients with a NK, MDS patients with 1 cytogenetic aberration, MDS patients with 2 cytogenetic aberrations, and MDS patients with a CK (highest) (Figure 1D). Additionally, we conducted a correlation analysis between recurrently mutated genes in MDS and *ONECUT3* levels (Supplemental Table 5) and noted a correlation between high expression levels of *ONECUT3* and mutant *PHF6* (*P* = 0.029), WT *IDH2* (*P* = 0.018), and WT *KMT2D* (*P* = 0.033).

To further assess whether high ONECUT3 protein levels correlated with CK, we generated the rabbit anti-ONECUT3 antibody with high specificity (Supplemental Figure 1, C and D). First, we applied Western blot analysis to detect endogenous ONECUT3 expression in BM mononuclear cells from patients with MDS. ONECUT3 protein expression was detected at high levels in CK and at relatively lower levels in NK, followed by levels in healthy volunteers (Figure 1E). In addition, flow cytometry showed a high abundance of ONECUT3 in BM hematopoietic stem/progenitor cells (HSPCs) (CD34^+^CD38^–^ and CD34^+^CD38^+^) from patients with MDS with an aberrant karyotype compared with its expression in patients with MDS with a NK and the healthy donor group (Figure 1F and Supplemental Figure 1E). These data indicated that a high abundance (protein and mRNA) of ONECUT3 was correlated with a CK in MDS.

### ONECUT3 OE leads to multinucleation and a CK independent of TP53.

**Figure 2:**
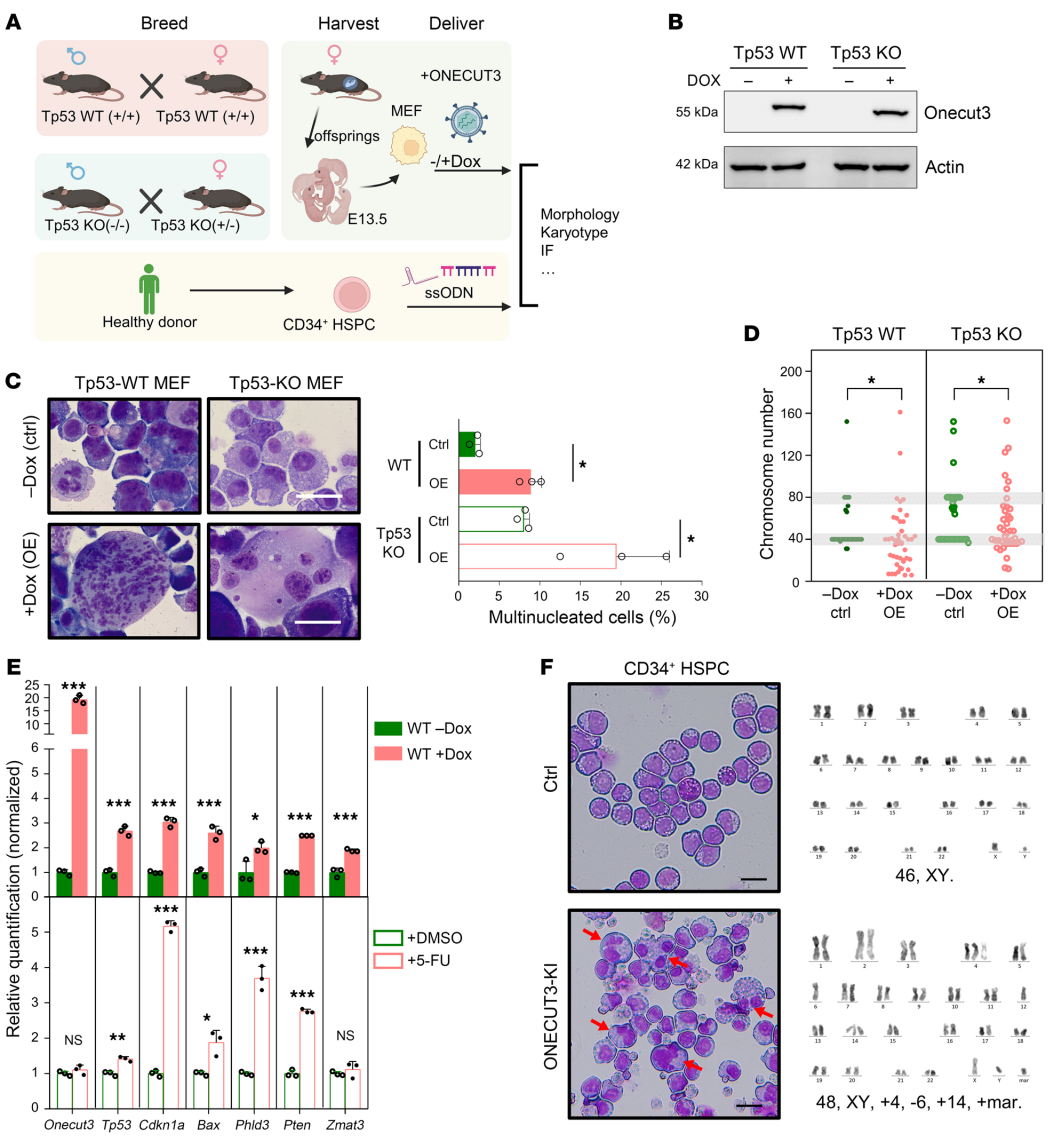
ONECUT3 OE leads to multinucleation and a CK independent of TP53. (**A**) Overview of experimental approach: WT TP53 embryos were acquired by breeding WT Tp53 male and WT Tp53 female mice. Tp53-KO embryos were acquired from breeding of Tp53-KO homozygotic males and Tp53-KO heterozygotic females. The E13.5 embryos were obtained from the above breeding, and MEFs were later isolated from these embryos, with *Tp53^+/+^* and *Tp53^–/–^* genetic backgrounds, respectively. HSPCs were obtained from a healthy donor and isolated to obtain CD34^+^ cells. Onecut3 expression was enforced by the Retro-X Tet-One system in the MEFs, and by ssODN in the human CD34^+^ HSPCs. Then we assessed the morphology, karyotype, and indirect IF. (**B**) Cell lysates were harvested after treatment with or without Dox (100 ng/mL) in both WT Tp53 and Tp53-KO MEFs for 48 hours and were blotted against anti-ONECUT3 antibodies. Actin was used as a loading control. (**C**) Representative images of WT Tp53 and Tp53-KO MEFs after 4 days of Dox treatment, followed cytospin and then Wright-Giemsa staining. Graph shows a comparative analysis of the multinucleated cell percentage (*n* = 3). (**D**) Chromosome number analysis in over 30 mitotic cells. Data points represent individual WT Tp53 and Tp53-KO MEFs. Conventional MEF chromosome numbers (*n* = 40 or 80; in grayscale) were considered as a benchmark; deviations were deemed anomalous. (**E**) Cells were either subjected to induction of ONECUT3 OE (upper panel) or to treatment with 380 μM 5-FU to induce *Tp53* expression or with DMSO for 24 hours (lower panel). (**F**) Analysis of morphology (left) and karyotype (right) for ssODN-mediated control and ONECUT3 expression in human CD34^+^ HSPCs. Red arrows indicate multinucleated cells. Scale bars: 100 μm. Error bars represent the SD. NS, not significant; *P < 0.05, **P < 0.01, and ***P < 0.001, by 2-tailed, paired Student’s *t* test (**C** and **F**–**H**) or χ^2^ test (**D**).

On the basis of the above clinical findings, we then investigated the effect of ONECUT3 OE on TP53-independent karyotype formation. To begin, WT Tp53 and Tp53-KO mouse embryonic fibroblasts (MEFs) were generated from the related E13.5 embryos obtained from heterozygotic breeding (Figure 2A). Then, enforced Onecut3 expression was induced by the tetracycline-inducible retroviral gene expression system (Retro-X Tet-One inducible expression system) in both WT Tp53 and Tp53-KO MEFs. After 48 hours of doxycycline (Dox) treatment, both WT Tp53 and Tp53-KO MEFs exhibited Onecut3 OE, indicating that inducible ONECUT3-overexpressing stable MEFs were successfully generated (Figure 2B). Importantly, ONECUT3 OE gave rise to more multinucleated variants, hyperdiploid cells, and aberrant chromosome numbers in both WT Tp53 and Tp53-KO MEFs when compared with those without Dox induction (which served as a control; Figure 2, C and D). Chromosome missegregation is known to activate the Tp53 signaling pathway in cells with an abnormal centrosome count (16, 17). As expected, we subsequently observed transcriptional upregulation of Tp53 and of its downstream targets in cells expressing Onecut3 with multinucleation. In contrast, Tp53 pathway activation by fluorouracil (5-FU) did not affect the expression of ONECUT3 (Figure 2E). These data suggested that ONECUT3 OE led to multinucleation and an aberrant karyotype independent of Tp53.

To confirm the phenotypes of the ONECUT3-mediated aberrant karyotype in human cell models beyond TP53, we increased ONECUT3 expression in primary human CD34^+^ HSPCs via a precise safe-harbor–targeted knockin technique using CRISPR/Cas9 (18) (Supplemental Figure 2A). Molecular analyses confirmed that targeted integration of ONECUT3 achieved up to 83% efficiency (Supplemental Figure 2, B and C), and the integration of the ONECUT3 region was confirmed. After 7 days of ONECUT3 OE in HSPCs, the cells displayed multinucleated variants, dysplasia, and aberrant karyotypes (Figure 2F). Additionally, the MDS-associated cell line MOLM13, expressing WT TP5, showed an intensive phenotype of multinucleation upon ONECUT3 induction (Supplemental Figure 2, D–F). Furthermore, upon introduction of ONECUT3 OE into the TP53-null acute myeloid leukemia (AML) cell line HL-60, we detected an increased percentage of double minutes formation in metaphase (Supplemental Figure 2G), which was possibly due to missegregated chromosomes and defects in chromatin bridge formation (19). These data indicated that ONECUT3 OE caused multinucleation and aberrant chromosomal structure in various human cell models, independent of TP53, suggesting that ONECUT3 might be a key driver of chromosomal abnormalities in MDS.

### ONECUT3 activates the chromosomal passenger complex components INCENP and CDCA8 through direct DNA binding.

**Figure 3:**
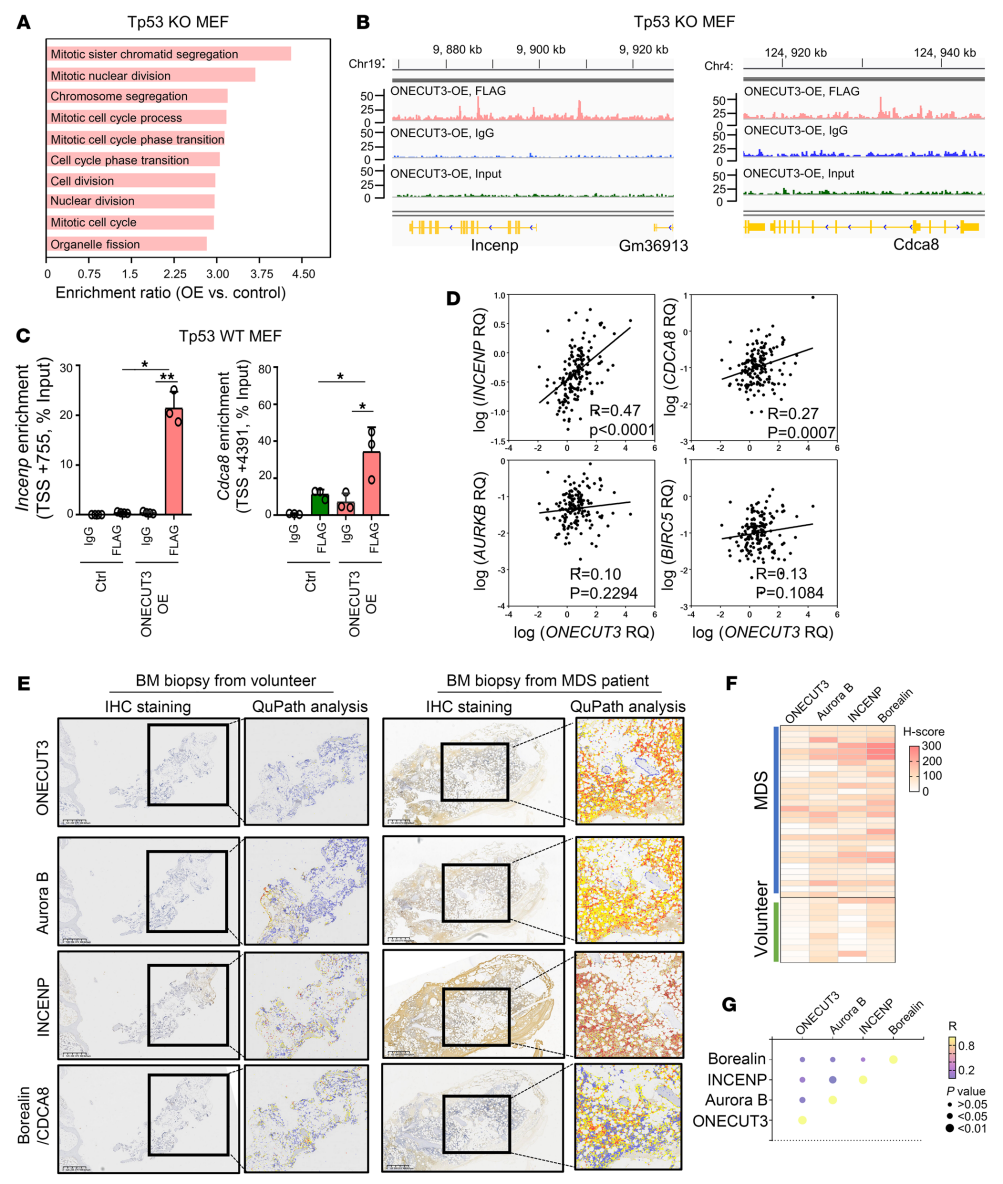
ONECUT3 activates the CPC components INCENP and CDCA8 through direct DNA binding. (**A**) RNA-Seq was conducted on the sample collected after 48 hours of Dox (100 ng/mL) treatment (to induce ONECUT3 OE) compared with no Dox treatment (control) in Tp53-KO MEFs. Gene sets, which were significantly enriched (FDR ≤ 0.05) in the Onecut3-overexpressing cells, are listed according to the normalized enrichment score (NES), using the GO Biological Process program. The top 10 biological processes are shown. Colored bars represent the enrichment ratio. (**B**) Integrative Genomics Viewer (IGV) tracks by ChIP-Seq in Tp53-KO MEF depicts the normalized density profile on *Incenp* and *Cdca8* gene loci. Onecut3 binds at 3 loci (the promoter, intron, and distal regions) of the *Incenp* gene, as well as at 2 loci of the *Cdca8* gene. (**C**) Enrichment of *Incenp* and *Cdca8* was found upon Onecut3 OE via ChIP-qPCR analysis in WT Tp53 MEFs. TSS, transcription start site. (**D**) Correlation between mRNA levels of ONECUT3 and *INCENP*, *CDCA8*, *AURKB*, and *BIRC5* in 165 patients with MDS harboring WT TP53. (**E**) Representative images of immunohistochemical staining (ONECUT3, Aurora B, INCENP, and Borealin/CDCA8) of biopsy tissue from a volunteer (healthy donor) and a patient with MDS (the right black box was the magnified view of the indicated region in the left box). Single-cell color outlines indicate QuPath-analyzed expression (0, blue; 1+, yellow, 2+, orange, 3+, red). Scale bars: 625 μm (left) and ×2 magnification (right). (**F**) Heatmap shows the H-Score with a combination of the staining intensity and cell frequency in each sample. (**G**) Correlation of the abundance of ONECUT3 with Aurora B expression, as determined by IHC. The color and size of the dots indicate the correlation coefficient (*R*) and *P* values. RNA-Seq replicates = 3, ChIP-Seq replicates of OE-Input/IgG = 1, replicates of Ctrl/FLAG = 1, replicates of OE-FLAG = 3. The correlation coefficient (*R*) and *P* values from Pearson’s correlation tests are shown (**D** and **G**). *P < 0.05 and **P < 0.01, by 1-way ANOVA (**C**).

The findings observed above prompted us to investigate the potential effects of ONECUT3 on chromosomal stability. To study how ONECUT3 contributes to chromosomal instability, we used MEFs with Onecut3 OE for mechanistic experiments because of the feasibility. As such, RNA-Seq and ChIP-Seq were concurrently performed in Onecut3-OE and its control on Tp53-KO MEF cells. GO annotation and GSEA of RNA-Seq data showed that sister chromosome exchange separation, mitotic nuclear division, and chromosome segregation were upregulated (Figure 3A and Supplemental Figure 3, A and B). Binding and Expression Target Analysis (BETA) Suite software (http://cistrome.org/BETA/) was used to integrate data from both RNA-Seq and ChIP-Seq. Although Onecut3 had both activating and repressing transcriptional functions, its activating function was shown to dominate other genes (Supplemental Figure 3, C–E). The top 10 directly activated target genes predicted by BETA include *Incenp* and *Cdca8* (Supplemental Figure 3F). ChIP-Seq tracks showed binding of Onecut3 to the promoter, intron, and distal regions of the *Incenp* gene and the gene body of the *Cdca8* gene (Figure 3B). This finding was also validated in WT Tp53 MEFs (Figure 3C and Supplemental Figure 3G). Accordingly, OE of Onecut3 could promote the transcription of *Incenp* and *Cdca8* in MEFs.

Coincidentally, Incenp and Borealin (the protein product encoded by *CDCA8*) are critical components of chromosomal passenger complex (CPC). Using in vivo data to corroborate the conclusion from the MEF models that Onecut3 activated the transcriptional induction of Incenp and Cdca8 expression, we conducted quantitative real-time PCR using BM tissue samples from patients with MDS (*n* = 165). We observed a positive correlation for transcriptional levels of *ONECUT3* with *INCENP* (*R* = 0.47, *P* < 0.01) and *CDCA8* (*R* = 0.27, *P* < 0.01) in patients with MDS (Figure 3D). Moreover, we performed immunohistochemical staining and unbiased quantitative analysis (QuPath) at the single-cell level in BM cells from volunteers (*n* = 10) and patients with MDS (*n* = 30). Indeed, BM from patients with MDS showed higher expression of ONECUT3, Aurora B, INCENP, and Borealin/CDCA8 than did BM samples from volunteers (Figure 3, E and F). Intriguingly, although no statistical difference was observed in the correlation between *AURKB* and *ONECUT3* at the RNA level, it was noteworthy that ONECUT3 had a robust correlation with the tissue protein level of Aurora B and a moderate correlation with INCENP or Borealin (also known as CDCA8) (Figure 3G and Supplemental Figure 3H). These data supported the relevance of ONECUT3 and its target components of the CPC in patients with MDS.

### OE of ONECUT3 leads to dysregulation of the CPC and mitotic defects.

**Figure 4:**
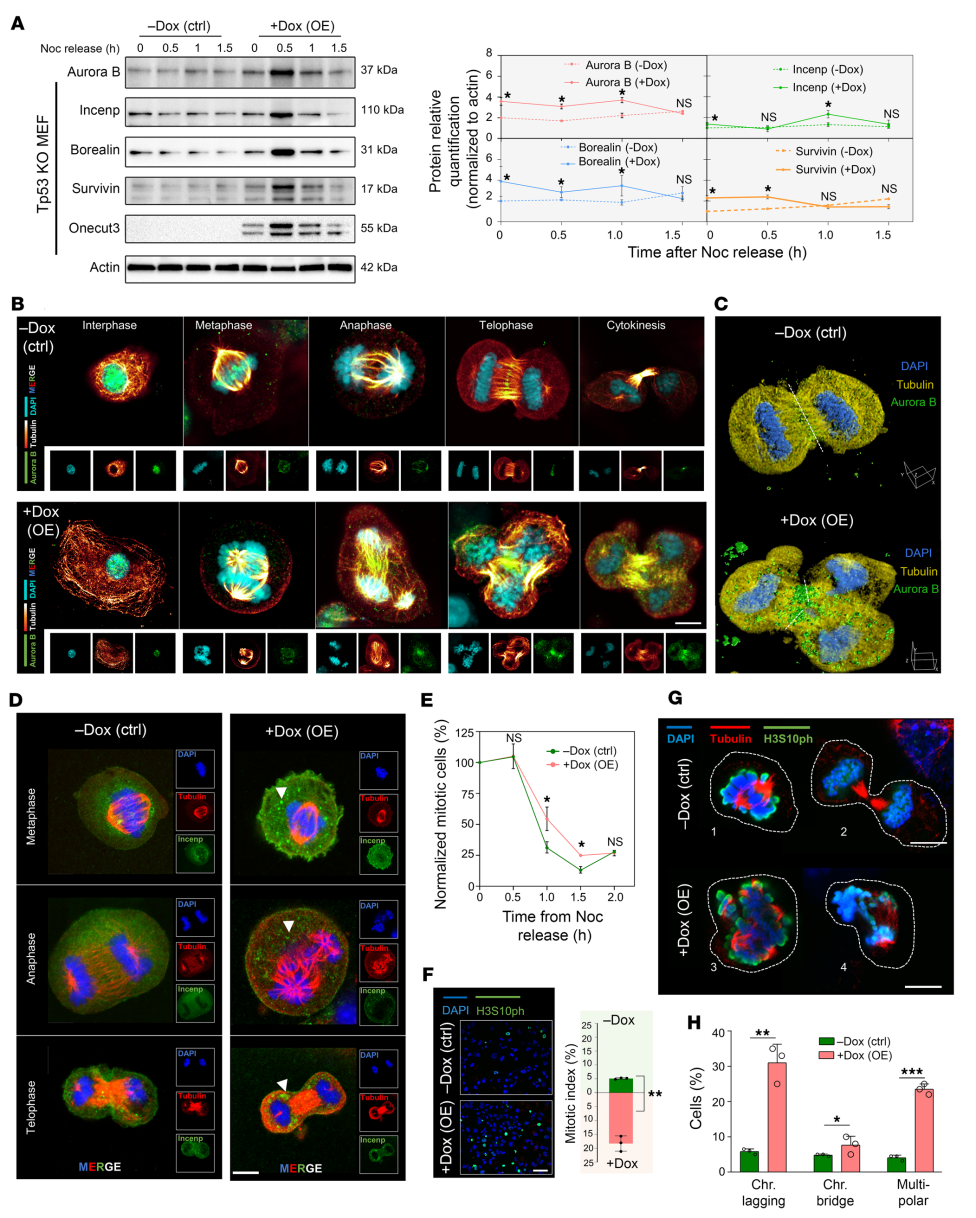
OE of ONECUT3 leads to dysregulation of the CPC and mitotic defects. (**A**) Time course of Western blot analysis of CPC components. Left: Control (–Dox) and ONECUT3 OE (+Dox) Tp53-KO MEFs were treated with nocodazole (Noc) (75 ng/mL) for 15 hours and released at the indicated time points. Cell lysates were blotted with antibodies against Incenp, Borealin, Survivin, and Aurora B. Protein levels were comparatively analyzed at the indicated time points, with gray values determined using ImageJ software (NIH). (**B**) Representative confocal images of the coimmunostaining against DAPI (blue), tubulin (yellow), and Aurora B (green) in each mitotic phase. (**C**) 3D model generated by Huygens, based on photos from *Z*-stack scanning. White dashes indicate the putative cleavage furrow. (**D**) Cells were immunolabeled at the indicated phases with α-tubulin (red) and Incenp (green) antibodies. White arrows indicate the differential localization of Incenp in Onecut3-overexpressing cells compared with the control. (**E**) Flow cytometry was performed to determine the fraction of mitotic cells, using H3S10ph and propidium iodide costaining following nocodazole release, normalized to the initial point (*t* = 0) for each time point. (**F**) Following Dox treatment and subsequent nocodazole treatment and washout, DAPI (blue) and H3S10ph (green) coimmunostaining was performed. Shown is a representative confocal image 1 hour after nocodazole release. Graph shows the mitotic index, evaluated 1 hour after nocodazole release. Randomly counted the number of H3S10ph^+^ cells from 62–120 cells/slide; *n* = 3. (**G**) Costaining with α-tubulin (red), H3S10ph (green), and DAPI (blue). White dashes outline control cells (outlined areas 1 and 2) and Onecut3-overexpressing cells (outlined areas 3 and 4). (**H**) Percentages of cells showing chromosome lagging, chromosome bridging, and multipolarity (*n* = 3). Scale bars: 11.6 μm (**B** and **D**), 10 μm (**C** and **G**), 100 μm (**F**). *P < 0.05, **P < 0.01, and ***P < 0.001, by 2-way ANOVA and 2-tailed, paired Student’s *t* test (**E**, **F**, and **H**).

The CPC, which consists of INCENP, Borealin, Survivin, and Aurora B kinase, derives its name from its characteristic dynamic localization during mitosis (20). We next examined whether ONECUT3 OE could affect the protein expression in the CPC. In order to enhance the enrichment of mitotic phases, MEFs were synchronized by nocodazole treatment (a reversible antimitotic agent) (21, 22). We observed an increase in Incenp, Borealin, Survivin and Aurora B expression in Onecut3-overexpressing cells during the immunoblotting assays after nocodazole release in Tp53-KO and WT Tp53 MEFs (Figure 4A and Supplemental Figure 4A). The immunofluorescence (IF) assay showed mislocalization of Aurora B at different mitotic phases, and the 3 daughter cells could be found in the Onecut3 OE groups (Figure 4B and Supplemental Figure 4B). The 3D model showed that Aurora B in the control group was localized to the midbody-like structure during telophase, whereas Aurora B exhibited diffuse cytosolic fluorescence upon Onecut3 OE (Figure 4C). We also noticed excessive aggregation of Incenp in Onecut3-overexpressing cells during the mitotic process (Figure 4D). Therefore, the above analysis demonstrated that a high level of Onecut3 expression resulted in an overabundance of CPC components.

The CPC performs various essential functions, including, but not limited to, the rectification of erroneous kinetochore-microtubule attachments, regulation of the spindle assembly checkpoint, and facilitation of cytokinesis, chiefly in maintaining chromosome stability (20, 23). We then explored whether OE of Onecut3 could disrupt spindle assembly and chromosome segregation. Onecut3 OE favored a relative delay of mitotic exit by flow cytometry and IF assay (Figure 4, E and F, and Supplemental Figure 4D). Interestingly, we observed a higher rate of chromosome bridge formation and multipolar spindle formation in Onecut3-overexpressing MEFs (Figure 4, G and H, and Supplemental Figure 4B). Notably, a higher occurrence of unaligned chromosome and multipolar spindle formation was evident in ONECUT3-overexpressing CD34^+^ HSPCs (Supplemental Figure 5, A and B).

In order to ascertain the potential causative relationship between Onecut3 OE and cytokinesis defects resulting from upregulation of its downstream target Incenp, we conducted the genetic manipulation of Incenp in both WT Tp53 and Tp53-KO MEFs and found that Incenp OE phenocopied the mitotic defects of Onecut3 OE, albeit with a slightly diminished multipolarity (Supplemental Figure 5, C–E). This discrepancy might be attributed to the upregulation of *Cdca8* (encoding Borealin), which plays a crucial role in maintaining the stability of bipolar spindles (24). MEFs were then transfected with a siRNA against Incenp and treated with Dox to synchronize the expression of Onecut3 (Supplemental Figure 6, A–C). As demonstrated in Supplemental Figure 6, D and E, the –Dox control (Ctrl) with Incenp-knockdown-only (siIncenp) group showed increased unaligned chromosomes and multipolarity in both WT Tp53 and Tp53-KO MEFs. These data points were in line with previous findings that INCENP knockdown (KD) failed to facilitate CPC recruitment to the centromere during pro-/metaphase, hindering midbody formation and the completion of cytokinesis so as to result in multinucleation and polyploidy (25, 26). However, in the +Dox (Onecut3 OE) with si-Incenp group (Supplemental Figure 6, D and E), MEFs exhibited fewer unaligned chromosomes compared with the –Dox (Ctrl) with si-Incenp group, indicating that Incenp KD conferred a partial rescue of the mitotic abnormalities induced by Onecut3 OE. As uncovered in previous literature, KD of human INCENP (hINCENP) reduced the abundance of the CPC subunits, but the normal CPC phenotype was rescued by full-length ectopic INCENP (25, 27, 28). Taken together, these results indicated that ONECUT3 might be a key factor causing cytokinesis failure and defective chromosome segregation through dysregulation of its target CPC components.

### ONECUT3-overexpressing cells present multidrug resistance, which could be mitigated by targeting of the ONECUT3/CPC axis.

Given that chromosomal instability confers multidrug resistance in cancer (29, 30), we sought to investigate whether ONECUT3 expression would affect the sensitivity of cells to chemotherapeutic drugs. Indeed, Onecut3-overexpressing cells were less sensitive to doxorubicin (the topoisomerase II inhibitor), vincristine (the microtubulin inhibitor), and decitabine (the DNA methyltransferase inhibitor) in vitro (Supplemental Figure 7, A and B). As ONECUT3-overexpressing cells attenuated chemosensitivity, we explored whether the compounds targeting the ONECUT3/CPC axis could alleviate chemoresistance. At present, small-molecule compounds specifically targeting the ONECUT3 protein remain largely unknown. To minimize the region of ONECUT3 protein for the virtual screening, we first sought to map the DNA-binding domain by generating truncated ONECUT3 plasmids (Supplemental Figure 8A). As a result, ONECUT3 occupied the locus of INCENP and CDCA8 mainly by the homeobox domain (HOX) domain (Supplemental Figure 8, B–E). These data suggested that the HOX domain of ONECUT3 served as the DNA-binding domain. Then, we applied virtual screening of the compound library (over 500,000 compounds) based on the DeepMind prediction of the human ONECUT3 protein structure (Supplemental Figure 9, A and B).

**Figure 5:**
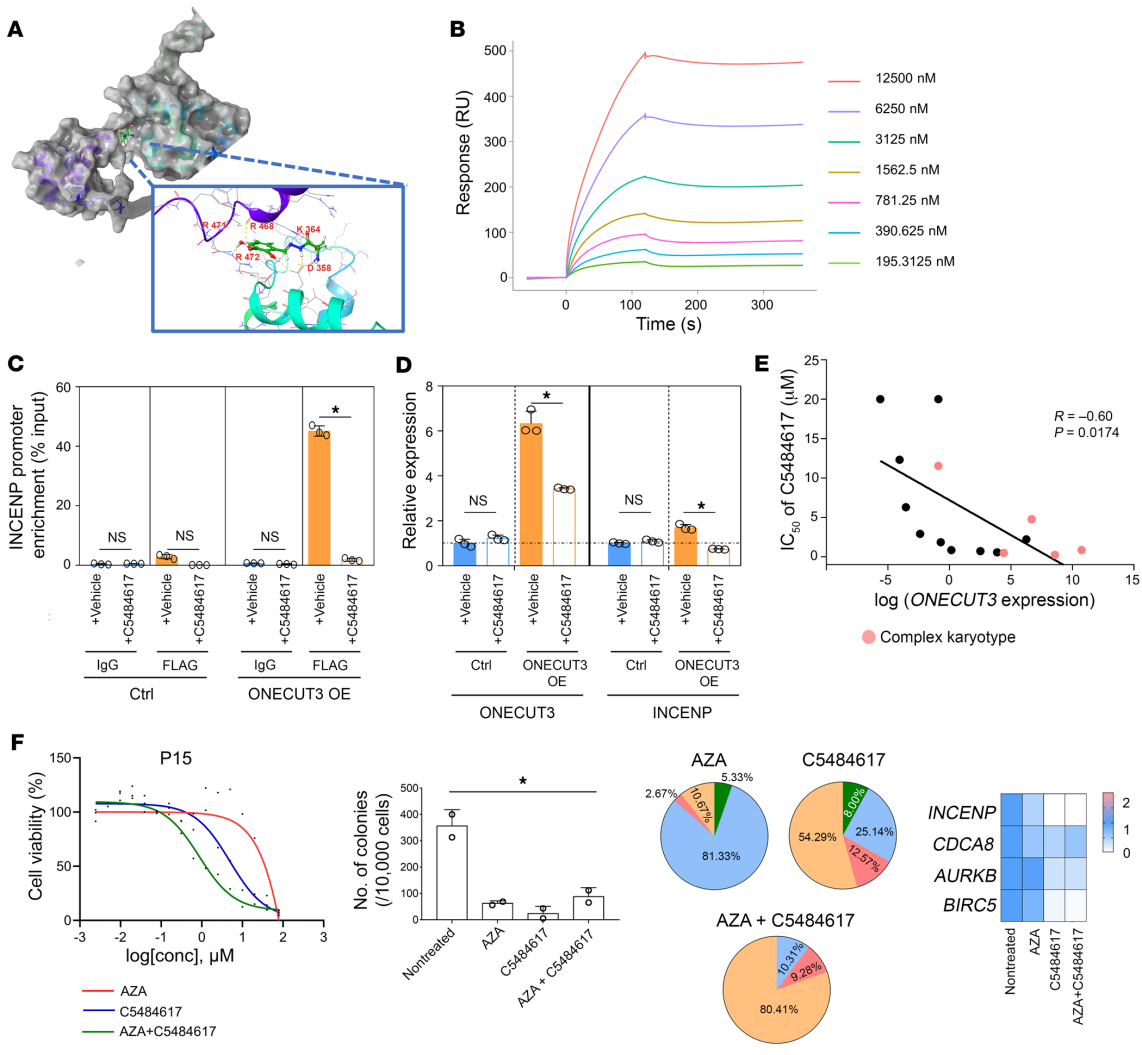
ONECUT3-overexpressing cells show multidrug resistance that could be mitigated by targeting the ONECUT3/CPC axis. (**A**) Docking modeling of the ONECUT3 HOX domain with the C5484617 compound using the Glide module. (**B**) SPR results show the binding of C5484617 to purified ONECUT3 protein in a dose-dependent manner. (**C** and **D**) HEK293T cells were transiently transfected with control (MSCV-vector) and ONECUT3 OE (MSCV-ONECUT3-WT) constructs for a duration of 48 hours. Following this, ChIP-qPCR (**C**) and qPCR (**D**) assays were performed after an additional 36-hour treatment with either vehicle (DMSO) or 2.5 μM C5484617. Graph illustrates the comparative analysis of the enrichment of the INCENP promoter (**C**) and mRNA levels of *ONECUT3* and *INCENP* in different treatment groups (**D**) (*n* = 3). (**E**) Correlation between mRNA levels of *ONECUT3* and the IC_50_ of C5484617 in MDS specimens (*n* = 15). Pale red dots indicate the MDS patient with a CK. (**F**) Representative data from the BM mononuclear cells from a patient with MDS following 48 hours of drug treatments (azacitidine, C5484617, azacitidine with C5484617): cell viability (left), quantification of clone numbers (middle left), proportions of morphological changes (pie chart at middle right, see also Supplemental Figure 11C), and assessment of *ONECUT3*, *INCENP*, *CDCA8*, *AURKB*, and *BIRC5* mRNA expression levels (right). *P < 0.05, by 2-tailed, paired Student’s *t* test (**C** and **D**) and Pearson’s correlation test (**E**).

The top 20 high-affinity compounds were identified according to the molecular docking score (Supplemental Table 6). The C5484617 compound was one of the lead hits that was available (Figure 5A). To further investigate the compound’s mechanism of action, we performed a direct binding assay by exposing purified recombinant ONECUT3 protein to C5484617 on a biosensor chip utilizing surface plasmon resonance (SPR) technology. The results demonstrated that the lead compound C5484617 bound to the ONECUT3 protein in a dose-dependent manner (Supplemental Figure 9C) with good affinity, as evidenced by the values of association rate constant (Ka) (1/ms) = 1,321, dissociation rate constant (kd) (1/s) = 8.926 × 10^–5^, and equilibrium dissociation constant (KD) = 6.755 × 10^–8^ (M) (Figure 5B). Liquid chromatography with tandem mass spectrometry (LC-MS/MS) screening following the unbiased drug affinity–responsive target stability (DARTS) assay confirmed ONECUT3 as one of its identified targets (Supplemental Figure 9D and Supplemental Table 7). Taken together, C5484617 possesses the ability to directly bind to ONECUT3 in a dose-dependent manner, whereas the interaction between C5484617 and ONECUT3 might detectably depress the transcriptionally active site(s) of ONECUT3.

We next explored the pharmacodynamic actions of C5484617 on ONECUT3. As shown in Figure 5, C and D, C5484617 reduced ONECUT3’s binding affinity to the INCENP promoter, and *INCENP* transcript levels were reduced by approximately 50% in ONECUT3-overexpressing cells, whereas C5484617 did not downregulate *INCENP* transcript expression in the control group. Although we observed that C5484617 downregulated *ONECUT3* mRNA levels (Figure 5D), it did not alter ONECUT3 protein levels, as shown in Supplemental Figure 9E. To further investigate the binding sites of C5484617 on ONECUT3, we conducted site-directed mutagenesis on 4 specific sites based on the docking model: ONECUT3-D358A, K364A, K370A, and R472A. Among these sites, mutation at ONECUT3-D358 alone prevented C5484617 from altering the binding efficacy of ONECUT3 to the INCENP promoter (Supplemental Figure 9F), which was necessary for the induction of INCENP transcription. This suggested that C5484617 probably inhibited ONECUT3 function through direct occupancy at the D358 site. Taken together, the pharmacodynamic actions of C5484617 resulting in inhibition of the transcriptional activity of ONECUT3 might have occurred through direct binding to the D358 site, consequently reducing the expression of ONECUT3 downstream targets, such as INCENP.

Next, we investigated the effect on cell death of ONECUT3 activity inhibition with C5484617 in patients with MDS. Our findings revealed an inverse relationship between *ONECUT3* mRNA levels in 15 newly diagnosed MDS patients and the IC_50_ of C5484617 (Figure 5E). Additionally, we observed a synergistic effect when combining barasertib (Aurora B inhibitor) or C5484617 with azacitidine (Supplemental Figure 10A). Furthermore, the use of C5484617 could potentially reduce the required concentration of azacitidine (Supplemental Figure 10B). Moreover, we observed a decrease in colony formation capacity, an increase in the proportion of enlarged cells, and an increase in the number of dead cells in the group treated with C5484617 and azacitidine in comparison with other groups. As expected, the administration of C5484617 resulted in a decrease in the mRNA levels of *ONECUT3* and its downstream targets, including *INCENP*, *CDCA8*, and *BIRC5* (Figure 5F and Supplemental Figure 11, A–C). These findings implied that C5484617 hampered the transcriptional functionality of ONECUT3, leading to a reduction in the expression of its downstream targets and ultimately inducing cell death in MDS. Consequently, these results indicated that cells overexpressing ONECUT3 exhibited resistance to chemotherapy, which could be partially mitigated by compounds targeting the ONECUT3/CPC axis, broadening the therapeutic landscape for patients with MDS.

## Discussion

**Figure 6:**
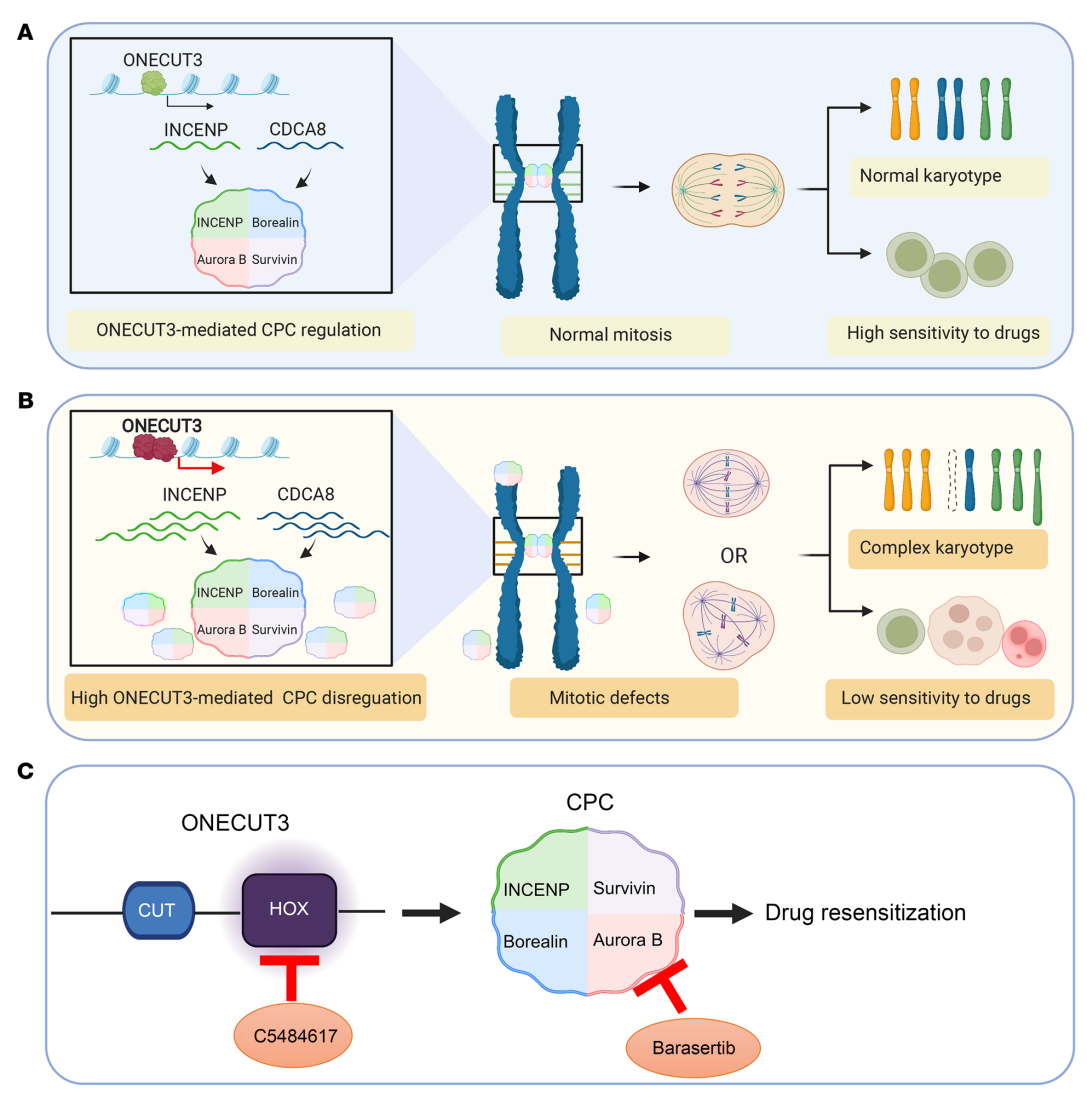
Schematic model depicting highly expressed ONECUT3 causing mitotic defects through upregulation of the CPC. (**A**) By modulating the levels of INCENP and CDCA8, ONECUT3 controlled the mitotic process, thereby ensuring the maintenance of a NK and enhancing sensitivity to drugs. (**B**) OE of ONECUT3 led to its binding to the genome of *INCENP* and *CDCA8*, resulting in the excessive activation of CPC component expression. Dysregulation of the CPC subsequently gave rise to mitotic defects, such as multipolar and chromosome missegregations, leading to the development of a CK and reduced sensitivity to drugs. (**C**) A lead compound, C5484617, was identified that functionally targeted the HOX domain of ONECUT3, inhibiting its transcriptional activity on downstream genes, and synergistically resensitized MDS cells to hypomethylating agents. It was worth noting that ONECUT3-overexpressing cells displayed chemoresistance, which could be partially alleviated through the use of compounds that target the ONECUT3/CPC axis, such as C5484617 or barasertib.

In this study, we found that high expression of the TF ONECUT3 was correlated with a CK in MDS. Mechanistically, ONECUT3 directly activated *INCENP* and *CDCA8*, leading to accumulation of the CPC components, multipolar division, and aberrant chromosome segregation. Moreover, the sensitivity of ONECUT3-overexpressing cells to chemotherapeutic drugs was weakened. However, the combination of hypomethylating agents (HMAs) and compounds targeting the ONECUT3/CPC axis could increase the sensitivity of drug-resistant MDS cells to HMA (Figure 6).

It is well established that the TF ONECUT3 belongs to the ONECUT family, an ancient HOX TF superfamily, which includes ONECUT1 (HNF6), ONECUT2, and ONECUT3 (31). ONECUT1 and ONECUT2 have been reported in tumors: highly active ONECUT1 can suppress the proliferation and metastasis of colorectal and lung cancer cells (32, 33), whereas ONECUT2 acts as a survival factor and a driver of prostate cancer (34). To date, research on ONECUT3 has focused on the physiological regulation of embryo development (13–15). The role of ONECUT3 in cancer is not well defined, and studies of ONECUT3 activity in myeloid neoplasms are limited. To our knowledge, this is the first report of its correlation with CK in MDS.

Here, we identified the target genes of TF ONECUT3. Surprisingly, the 2 components of the CPC were the direct targets of ONECUT3. When the localization or function of any CPC target component was disrupted, the remaining components could not be properly localized, leading to decreased Aurora B activity and cell division (35–37). INCENP and Aurora B kinases are overexpressed in various solid tumors (26, 38–40). The mislocalization and defective activity of Aurora B kinase were found in hyperdiploid B cell acute lymphoblastic leukemia (41). At present, data on myeloid neoplasms, especially MDS, are limited. Interestingly, Yoshida et al. reported that Aurora B and Survivin in BM CD34^+^ cells were highly expressed in patients with high-risk MDS and secondary AML (arising from MDS), suggesting that dysregulation of the CPC was closely related to high-risk MDS (42). However, the specific mechanism of the CPC in regulating mitosis in MDS remains unclear. This study found that the misexpression of CPC components controlled by ONECUT3 OE led to mitotic defects. Further research is needed to explore the specific role of ONECUT3 on sister chromatid segregation.

Even though chromosomal instability is a prominent feature in MDS, the previous studies focused on the relationship between *TP53* mutation and a CK (43, 44). Nevertheless, approximately 50% of patients with MDS with a CK do not harbor *TP53* mutations (7). Thus, identification of the mechanism of a CK in the remaining 50% of patients is urgently needed. In this study, we assessed the role of OE of ONECUT3 on the mitotic process on the basis of different *TP53* statuses. First, we identified the CK-associated TF ONECUT3 in patients with WT *TP53*. High ONECUT3 expression is also frequent and associated with a worse prognosis in different cancer data sets, e.g., data sets for lung adenocarcinoma (LUAD), lung squamous cell carcinoma (LUSC), adrenocortical carcinoma (ACC), and kidney renal clear cell carcinoma (KIRC). However, its upregulation was almost irrelevant with *TP53* mutations (data not shown). Second, we observed accumulation of ONECUT3 at its target genes, such as *INCENP* and *CDCA8*, and the resultant transcriptional induction (chromosome missegregation, drug resistance) in both WT Tp53 and Tp53-KO cell models. This suggested that ONECUT3, acting as a TF, was implicated in the dysregulation of INCENP and CDCA8 and the resultant drug resistance, independent of TP53 regulation. Third, it is well known that TP53 plays a critical role in limiting the propagation of multinucleation in human cells to maintain the diploid karyotype (45, 46). Studies by Teng et al. and Li et al. (47, 48) concluded that TP53 negatively regulates Aurora B via FBXW7. Diminished levels of TP53 in the ONECUT3 OE model might be one of the key reasons for the upregulation of Aurora B expression by approximately 2- to 4-fold compared with the approximately 1- to 1.8-fold increase observed in the TP53-WT model. Hence, the coexistence of TP53 KO and ONECUT3 OE resulted in a propagation of the entire CPC complex could be observed during cell-cycle progression. Furthermore, the upregulation and activation of TP53 might have been be a response to ONECUT3-induced chromosomal missegregation, as part of its role in mitotic surveillance. Intriguingly, even with an intact TP53 response, the ONECUT3-driven phenotypes of aberrant karyotypes and drug resistance persisted across multiple cell models, suggesting that ONECUT3 might be a potential therapeutic target for patients with MDS, particularly those with WT TP53.

The past decades have witnessed significant progress in MDS treatment with the advent of HMAs, Bcl-2 inhibitors, and so on (49–53). HMA or intensive induction chemotherapy remains the first-line therapy for patients with MDS, especially elderly MDS patients. Nevertheless, approximately 30%–50% of adult patients with MDS do not respond to HMAs (1, 54). Moreover, drug resistance might be an essential factor affecting the efficacy of chemotherapy and disease progression (55, 56). Therefore, it is important to explore different ways to minimize drug resistance. Although some ONECUT3-overexpressing cells had mitotic defects and died due to mitotic catastrophe, most ONECUT3-overexpressing cells survived (data not shown). More efforts are needed to explore the mechanisms of cell survival and chemoresistance under the stress of mitotic defects. In this study, we tried to harness the ONECUT3/CPC axis as an entry point to find possible target compounds by structure-based virtual screening to overcome chemoresistance. Our data indicated that C5484617 and the Aurora B inhibitor barasertib could reduce the resistance of cells to azacitidine, but in vivo experiments are still needed for further proof. At the same time, the clinical phase I/II trials of barasertib in acute myeloid leukemia showed that the inhibition of CPC components had an encouraging improvement in the complete response (CR) rate compared with low-dose cytosine (LDAC), further supporting the feasibility of targeting ONECUT3 (57, 58).

In summary, the current study demonstrated that the CK-associated TF ONECUT3 dysregulated the CPC components in MDS, leading to mitotic defects and decreasing chemosensitivity. The lead compound (C5484617) or Aurora B inhibitor (barasertib) could help increase the sensitivity of MDS cells to HMA. We believe our findings have mechanistic implications and clinical relevance. The present investigation provides the foothold for further studies to identify the approaches for MDS patients with limited therapeutic options. Moreover, our findings might be implemented for other cancer types, given that a CK and genomic instability are hallmarks of cancer.

## Methods

### Sex as a biological variant.

Patients, adult mice (6–10 weeks old), and mouse embryos of both sexes were used unless otherwise stated. Sex was not considered as a biological variable in this study.

### MDS patient samples and cell lines.

BM samples were collected from HSPC donors (*n* = 31) and newly diagnosed MDS patients with WT TP53 (*n* = 165) according to the Declaration of Helsinki. Informed consent was obtained from all participants, and the procedures were approved by the IRB of the First Affiliated Hospital, School of Medicine of Zhejiang University. Primary MEFs were isolated from the WT Tp53 and Tp53-KO embryos. In addition, MOLM13, HL-60, HEK293T, and platinum-E (Plat-E) cells were authenticated. Details are available in the Supplemental Methods.

### Single-stranded oligodeoxynucleotide–mediated ONECUT3 expression in human CD34^+^ HSPCs.

To integrate *ONECUT3* targeting at the CCR5 locus (18), we constructed an EGFP linked to ONECUT3 expression cassettes driven by the PGK promoter and terminated by an SV40 polyA (pA). The EGFP-ONECUT3 cassette was flanked by sequence-homologous arms to *CCR5*, which was targeted by the sgRNA (5′-GCCCAGTGGGACTTTGGAAAT-3′). The EGFP-ONECUT3-pA sequence with CCR5 homolog arms on each side was chemically synthesized in high purity of single-stranded oligodeoxynucleotides (ssODNs) by GeneScript Inc. Sequences and maps of the relevant parts are available in Supplemental Figure 2A. To achieve efficient genome editing in human CD34^+^ HPSCs (Saily Biotechnology Co., Ltd.), the optimizing MaxCyte electroporation condition was used to introduce the CRISPR/Cas9 system. Semiconfluent cells (3–4 days after transfection) were harvested for genomic DNA extraction and/or FACS analysis. Details are available in Supplemental information.

### Identification of ONECUT3 direct targets.

RNA-Seq and ChIP-Seq were performed on Tp53-KO MEFs to identify target genes both bound to and regulated by Onecut3. Onecut3 expression was induced using the Retro-X Tet-One inducible expression system (gift from Kosei Ito, Nagasaki University, Nagasaki, Japan). RNA-Seq was performed using the NextSeq 550 platform (Illumina). The enriched biological processes regulated were identified by gene ontology (GO) annotation and gene set enrichment analysis (GSEA). ChIP-Seq was performed using the HiSeq 2500 platform (Illumina). Model-based analysis of ChIP-Seq (MACS) was used to identify ChIP-Seq binding peaks, and edgeR25 was used to identify differentially expressed genes associated with OE of Onecut3. BETA was used to integrate data from both RNA-Seq and ChIP-Seq to predict the activating or repressive function, infer the direct target genes, and identify the motif of Onecut3 (59).

### Indirect IF and confocal microscopy.

Cells were synchronized by 75 ng/mL nocodazole for 15 hours and fixed with 4% paraformaldehyde for 30 minutes. Next, the cells were permeabilized with 0.5% Triton X-100 for 10 minutes and blocked with 3% BSA for 30 minutes. Cells were then incubated with primary antibodies in the antibody buffer (PBS plus 3% w/v BSA plus 0.05% Tween 20 plus 0.04% sodium azide) at 37°C for 1 hour and with fluorophore-conjugated secondary antibodies for an additional hour. Slides were mounted with Vectashield containing diamidino-2-phenylindole (Vector Laboratories). The images were acquired on a TCS SP8 Confocal Laser Scanning Microscope (Leica).

### Protein structure prediction and drug virtual screening.

The 3D structure of the human ONECUT3 protein was modeled using the AlphaFold Monomer, version 2.0, pipeline (60). Then, Prime and Protein Preparation Wizard in Schrodinger (Schrodinger Inc.) was applied to fix the missing residue structures and optimize the conformation of the protein. Subsequently, a sitemap module was used to detect putative binding sites of the protein. Chemicals from Hit2Lead (Chembridge) were first screened by a structure-based strategy and prepared using the Ligprep module. Glide with the standard/extra precision mode was used for virtual screening.

### SPR.

The purified ONECUT3 proteins were immobilized on a Series S Sensor Chip, CM5 (Cytiva), through amine coupling using the amine coupling kit (Cytiva). The samples flowed over the surface at 30 μL/min for a 120-second binding duration and a 240-second dissociation duration. The data obtained were analyzed using BIAcore T200 3.0 (Cytiva) with the Langmuir binding model.

### Statistics.

Unless otherwise noted, statistically significant data were assessed by unpaired, 2-tailed Student’s *t* test or Mann-Whitney *U* test. Tests for differences between more than 2 groups were performed using 1-way ANOVA with Bonferroni’s post hoc test, when applicable. The χ^2^ test or Fisher’s exact test was used for categorical variables as appropriate. OS was measured from the time of sample collection to the time of death from any cause; data on patients last known to be alive were censored. Survival curves were plotted using the Kaplan-Meier method with the log-rank test. The prognostic significance of each factor was determined using stepwise multivariate Cox regression models. Statistical analysis was conducted using GraphPad Prism 8.01 (GraphPad Software) or SPSS Statistics 25 (IBM Corp.). Data are presented as the mean ± SD or the median (range), and statistical significance was assessed at a *P* value of less than 0.05.

### Study approval.

Informed consent was obtained from all participants, and the procedures related to primary human samples were approved by the research ethics committee (IRB) of the First Affiliated Hospital, Zhejiang University School of Medicine (reference no. 2020-458). The collection of human BM samples complied with Declaration of Helsinki principles. Procedures related to animals were approved by animal experimental ethics inspection committee of the First Affiliated Hospital, Zhejiang University School of Medicine (reference no. 2019-432). Details are listed in the Supplemental Methods.

### Data availability.

RNA-Seq and ChIP-Seq data are available in the NCBI’s Gene Expression Omnibus (GEO) database (GEO GSE202694; https://www.ncbi.nlm.nih.gov/geo/query/acc.cgi?acc=GSE202694, secure token:ghahuoymhzkjjkn). All data are available from the corresponding author and are provided in the Supplemental Supporting Data Values file.

## Author contributions

YWL, GH, and HYT conceived the study. YWL, XMF, and GH designed the research. YWL, XMF, WL, WHX, SHZ, and HMZ performed research. CM, LY, LW, LYM, XPZ, YLR, JJ, and RZX acquired patient samples and provided facilities and other resources. YWL, XMF, and WL analyzed data. XPZ, YWL, WW, and WL performed statistical and bioinformatics analyses. YWL, XMF, and GH wrote the manuscript, and all authors reviewed and approved the manuscript.

## Supplementary Material

Supplemental data

Supporting data values

## Version 1 02/22/2024

In-Press Preview

## Version 2 04/15/2024

Electronic publication
