# Supplementary material for: Ectopic expression of the transcription factor ONECUT3 drives a complex karyotype in myelodysplastic syndromes
Source: J Clin Invest. 2024 Feb 22;134(8):e172468. doi: 10.1172/JCI172468 (PMC11014670; doi:10.1172/JCI172468)
Supplement: Supplemental data [file jci-134-172468-s183.pdf]

List of Supplemental Information for 'Ectopic expression of transcription factor ONECUT3 drives complex karyotype in Myelodysplastic Syndromes'

Supplemental Figure Legends and Figures P2-20
Supplemental Tables.. P21-35
Supplemental Material and Methods... P36-47
Supplemental References.. P47

SUPPLEMENTAL FIGURE LEGENDS AND FIGURES

Fig. S1

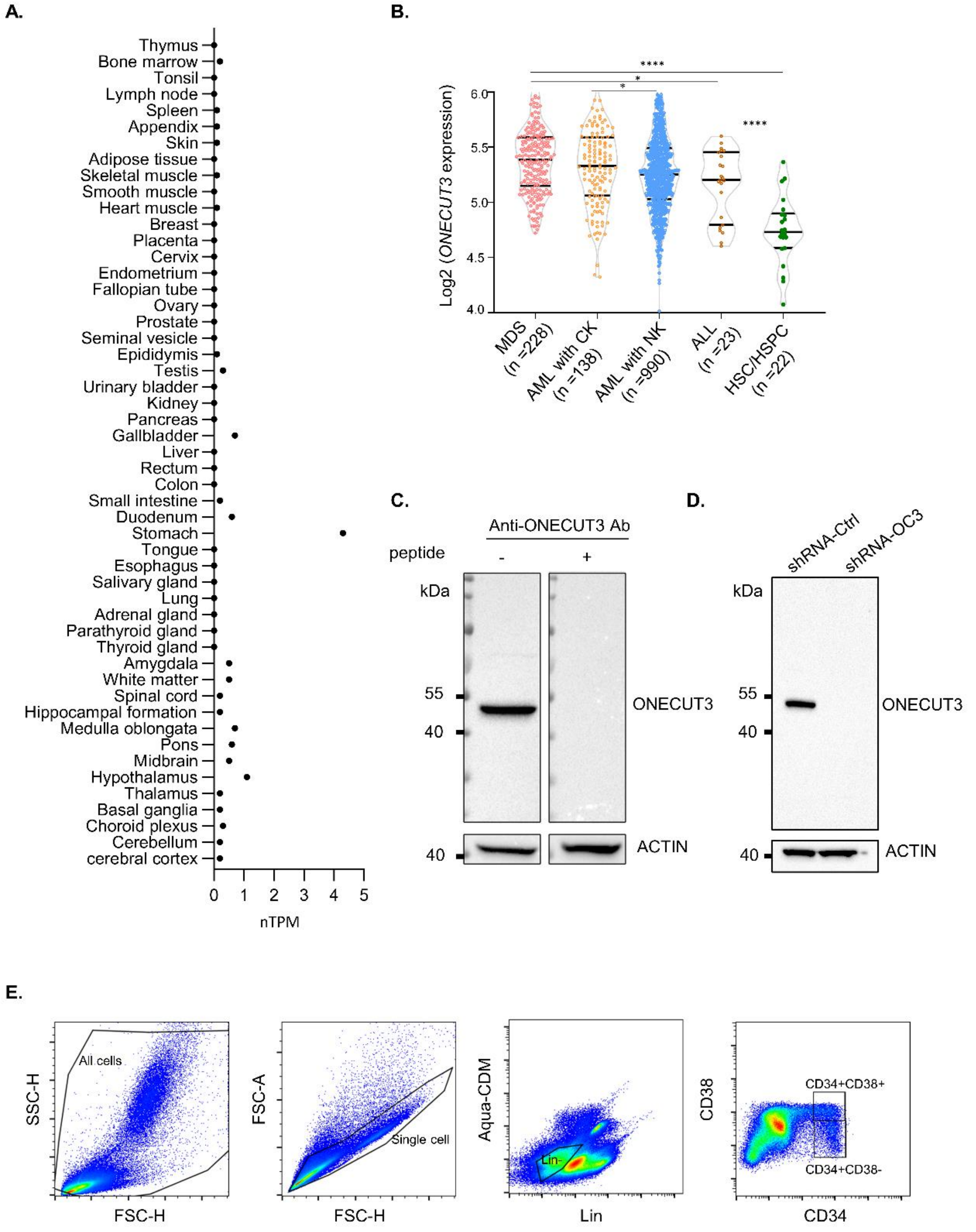

Supplemental Data Figure 1 (Related to Figure 1). A high level of Transcription factor ONECUT3 is correlated to CK in MDS.

A. The data is based on HPA RNA-seq tissue data from the Human Protein Atlas version 22.0. nTPM (normalized protein-coding transcripts per million), corresponds to the mean values of the different individual samples from each tissue. **B.** The expression of *ONECUT3* was generated from the dataset of GSE13159, GSE15434, GSE61804, GSE14468, and The Cancer Genome Atlas (TCGA). Data has been batch corrected. AML with CK contains: AML with Complex, AML with Complex +other and AML with Complex+5q. HSC/ HSPC includes Hematopoietic stem cell (HSC), Multipotential progenitors (MPP), Common myeloid progenitor cell (CMP), Granulocyte monocyte progenitors (GMP), Megakaryocyte-erythroid progenitor cell (MEP). **C-D.** The validation of newly generated anti-ONECUT3 antibody. The cell lysate of the bone marrow (BM) mononuclear cells from one patient of complex karyotype was used for immunoblotting.1 μg homemade antibody (rabbit anti-human ONECUT3 antibody) was not blocked [(–) blocking peptide] and another 1 μg antibody that pre-incubated with the peptide [(+) 1 μg blocking peptide (aa 469-482)] for overnight (C). HEK293 cells were transiently transfected with pLKO-TET-ONECUT3. In addition, the cell lysate from ShRNA-Ctrl (-Dox) or shRNA-ONECUT3 (+Dox) for the immunoblotting was also performed to confirm the specificity of the rabbit anti-ONECUT3 polyclonal antibody (D). Loading control is ACTIN. **E.** Gating strategy for $Lin^{-}D34^{+}CD38^{-}$ stem cells and $Lin^{-}CD34^{+}CD38^{+}$ progenitor cells from volunteers and patients with MDS in Figure 1 F.

Fig. S2

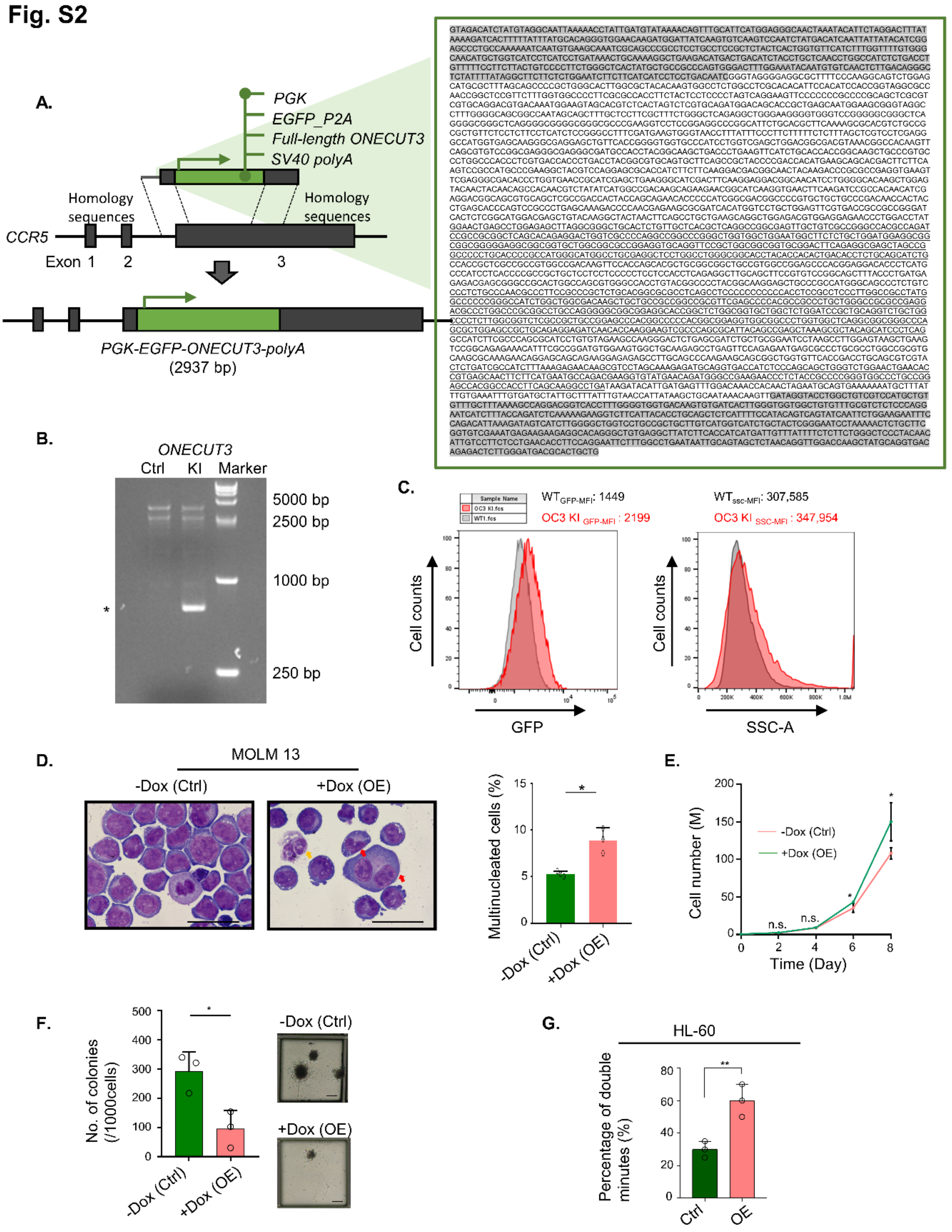

Supplemental Figure 2 (Related to Figure 2). Overexpressed ONECUT3 leads to multinucleation and complex karyotype independent from TP53

A. Schematics of CRISPR/CAS9-induced ONECUT3-targeted integration into *CCR5* locus. Left, the targeting ssODN was depicted as the PGK-EGFP-ONECUT3-SV40 polyA expression

cassette flanked by homology sequences (gray lines and boxes) to *CCR5*; right, sequence of ssODN of PGK-EGFP-ONECUT3-SV40 polyA cassette flanked by homology sequences. Gray-highlighted parts were homology sequences targeted *CCR5* exon3. The full-length of *ONECUT3* gene was underlined. **B**. Targeted integration of the indicated EGFP-ONECUT3 expression cassettes relative to wide-type and- EGFP+ hematopoietic stem/progenitor cells (HSPCs). The genotypes of control and ONECUT3-KI HSPCs were depicted by PCR. Ctrl: No band detected at 646 bp; ONECUT3-KI: band detected at 646 bp. **C.** Flow cytometry data showing EGFP+ cells 4 days after transduction with or without EGFP-ONECUT3 expressing. Two histograms showed the EGFP MFI (left) and SSC MFI (right) of control HSPC (gray) and EGFP+ HSPCs (red color). **D.** Morphology of MOLM13 cells after the eight days of Doxycycline induction was conducted via cytospin, followed by Wright-Giemsa staining. Left, the representative figures; red arrows indicate the multinucleated cell; right, comparative analysis of multinucleated cell percentage in control (-Dox) and ONECUT3-OE (+Dox) on MOLM13 cells; n=3. **E.** Cell growth was shown in the absence (control) or presence (ONECUT3-OE) of Doxycycline. The cell number was counted every two days. **F.** CFU-assay in methylcellulose after ONECUT3-OE or control in MOLM13 for 14 d. Left, the comparative analysis of CFU colony number; right, representative colonies are shown. **G.** HL-60 R-band chromosome analysis. The percentage of double minutes in 10 metaphases in control and ONECUT3-OE. Scale bar, 10 μm (D), 100 mm (F). Statistical analysis was performed using a two-tailed paired Student t-test (D, F, and G); n. s. not significant, *P <0.05, **P <0.01.

Fig. S3

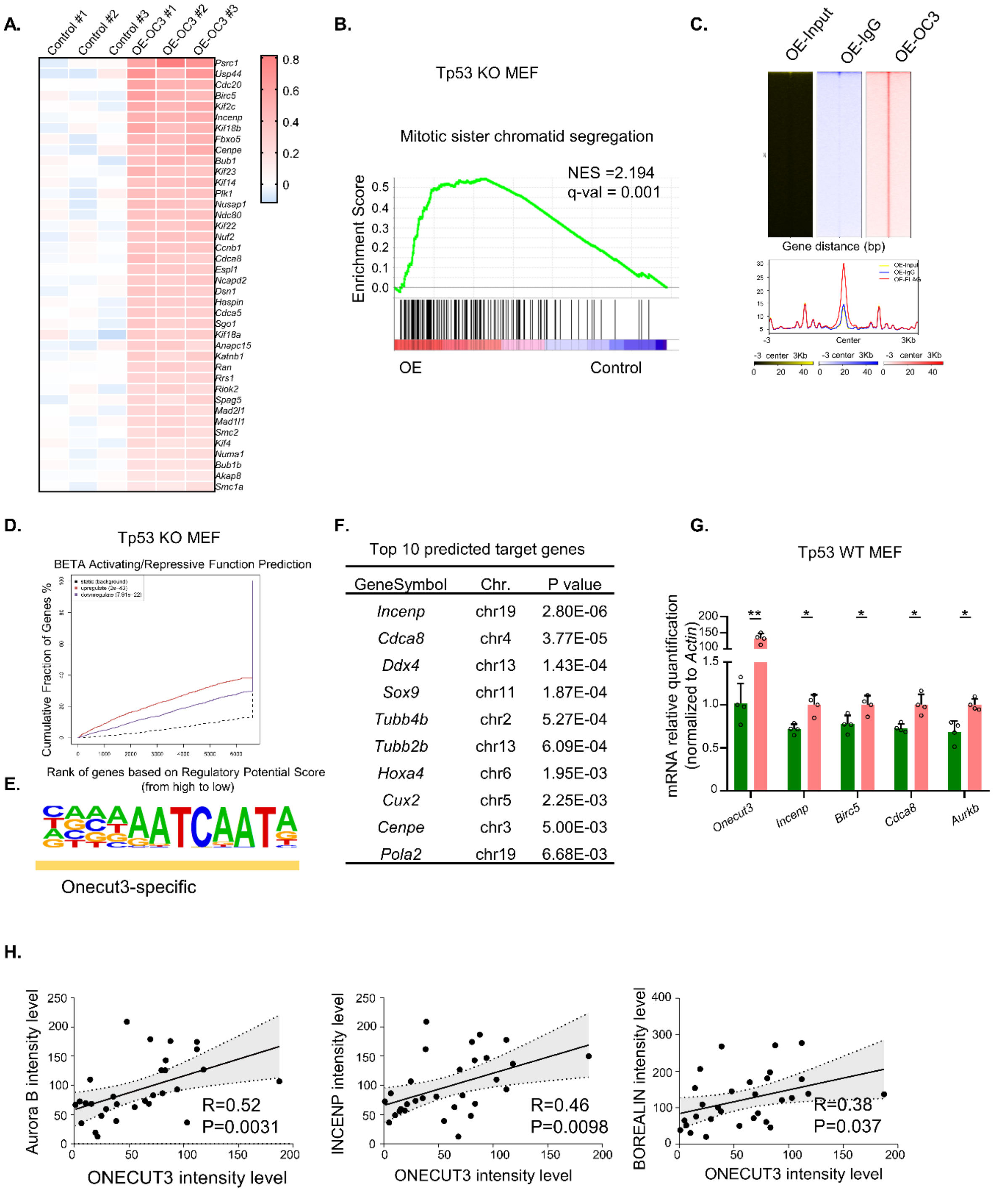

Supplemental Figure 3 (Related to Figure 3). ONECUT3 activates Chromosomal Passenger Complex components INCENP and CDCA8 through direct DNA binding.

A. RNA-seq was conducted on the sample collected after 48 h of Doxycycline (100 ng ml^{-1}) induction (Onecut3-OE) compared to without Doxycycline induction (control) in Tp53-KO MEF.

The heatmap showed the top 40 leading-edge genes from the GSEA of Onecut3-OE-induced genes. Expression values are represented as colors (pink-high, white-moderate, light blue-low). **B**. GSEA plot showing the ranked Onecut3-OE-induced genes in Tp53-KO MEF (related to Figure 3A). **C.** Distribution map of Onecut3 binding loci in ChIP-seq. **D.** BETA suit found Onecut3 in Tp53-KO MEF had both activating and repressing functions, while it dominated to activate other genes. **E.** Top binding motif in Onecut3-specific peaks. **F**. The integrative assays of RNA-seq and ChIP-seq were conducted by Binding and Expression Target Analysis (BETA). The top 10 predicted target genes are listed in the table. **G.** The enrichment of *Incnep* and *Cdca8* was found upon Onecut3-OE via quantitative PCR analysis in Tp53-WT MEF. **H**. The correlation of the IHC intensity level (H-Score) of ONECUT3 and Aurora B, INCENP or Borealin/CDCA5 in MDS biopsy tissue. The black line represents the regression curve derived from Pearson correlation analysis. The shaded area in the plot indicates the confidence interval (95%). Statistical analysis was performed using a two-tailed paired Student t-test (G.) or Pearson correlation tests (H.); n. s. not significant, *P <0.05, **P <0.01.

Fig. S4

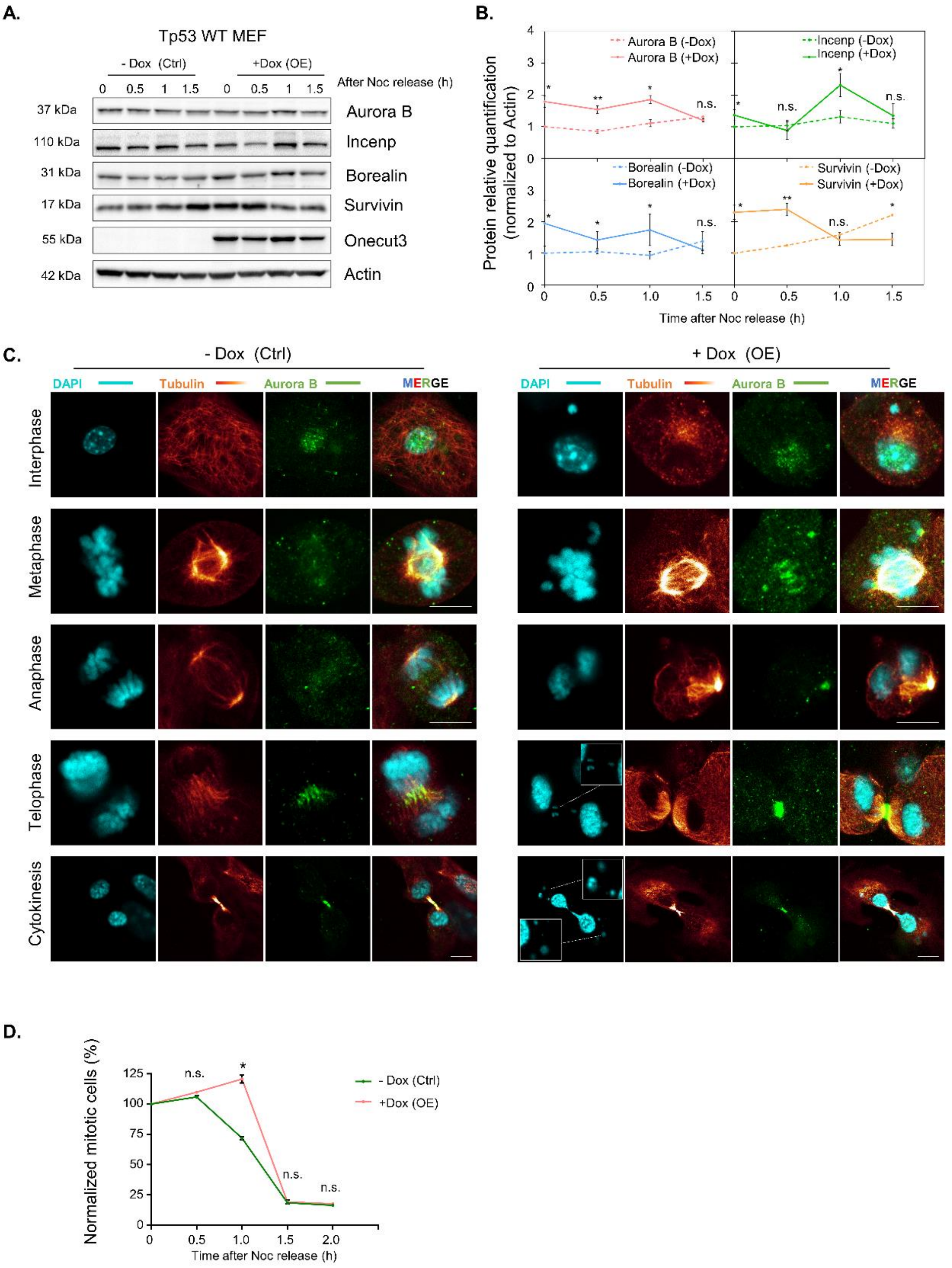

Supplemental Figure 4 (Related to Figure 4). Overexpression of ONECUT3 leads to dysregulation of Chromosomal Passenger Complex and mitotic defects.

A. Time course of Western blot analysis of CPC components. The control (-Dox) and Onecut3-OE (+Dox) on Tp53-WT MEF were treated with Nocodazole (75 ng ml^{-1}) for 15 hours, and

Nocodazole was washed out at the indicated time points. These cell lysates were then harvested for immunoblotting against the antibodies of CPC components (Incenp, Borealin, Survivin, and Aurora B). **B.** The comparative analysis of total protein levels at the indicated time points of nocodazole release: grey values were analyzed using ImageJ, and the ratio to Actin as a percentage was calculated relative to t = 0 for each time point. **C.** Representative confocal images of the co-immunostaining against DAPI (blue), Tubulin (yellow), and Aurora B (green) in each mitotic phase. **D.** After the nocodazole release, cells were fixed, and the fraction of mitotic cells was determined by FACS according to phospho-histone H3 staining and normalized to t = 0 h for each time point. Percentage control vs. Onecut3-OE is indicated.

Fig. S5

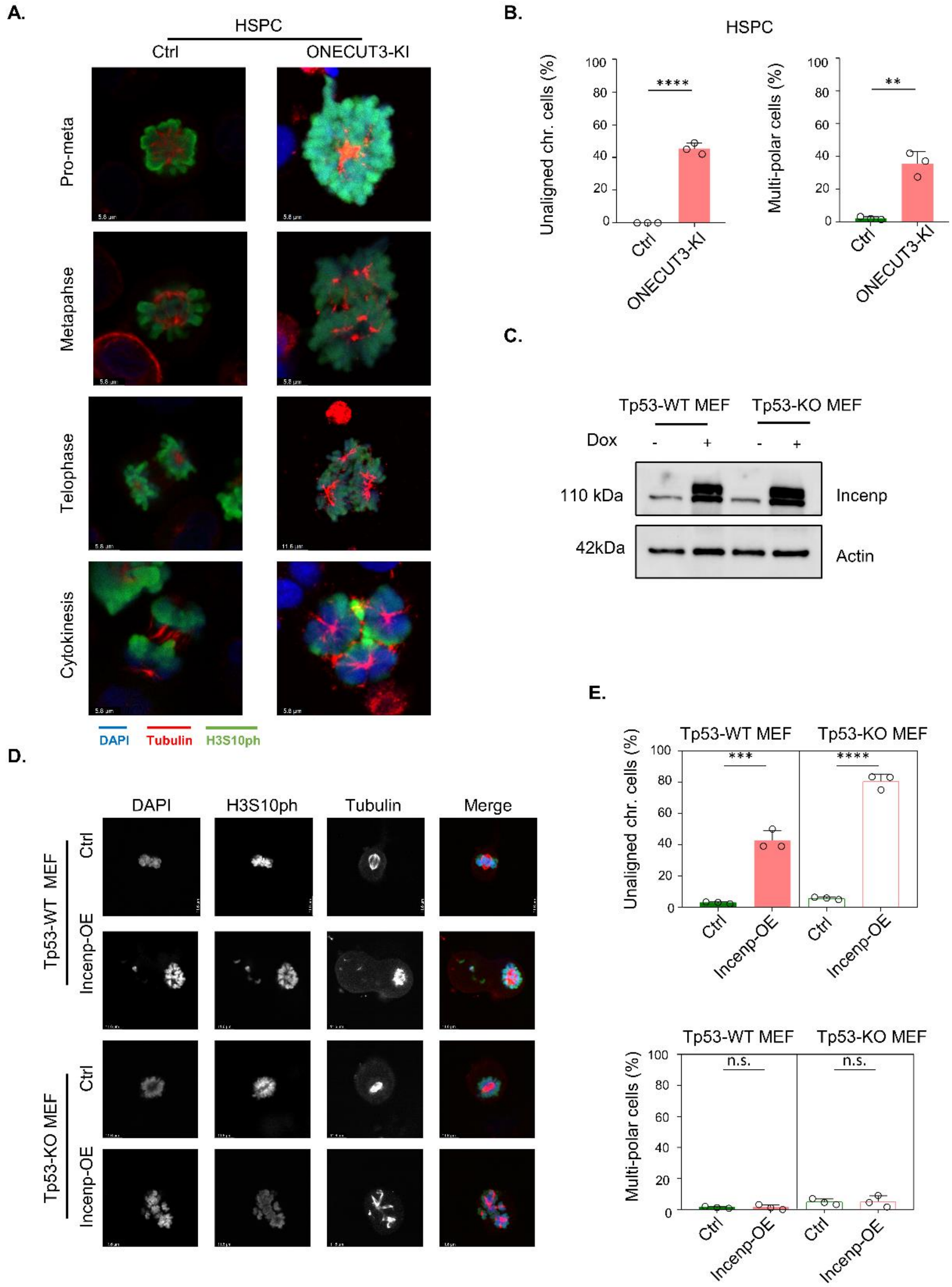

Supplemental Figure 5 (Related to Figure 4). Overexpression of ONECUT3 leads to the mitotic defect through the misexpression of CPC components.

A. The confocal was conducted after 7 days in HSPC and 15 hours of nocodazole treatment followed by PBS washout. The representative co-immunostaining image against DAPI (blue),

H3S10ph (green) and Tubulin (red) in each mitotic phase. **B**. The percentage of unaligned chromosomes and multi-polar cells in control and ONECUT3-KI HSPC were counted; n=3. **C.** The cell lysates were harvested after the treatment with or without 48h Doxycycline (100 ng ml^{-1}) to induce Incenp over-expression in Tp53-WT and Tp53-KO MEF and were blotted against anti-Incenp antibodies. Loading control is Actin. **D**. The representative co-immunostaining image against DAPI (blue), H3S10ph (green) and Tubulin (red) of control or Incenp-OE in Tp53-WT and Tp53-KO MEF. **E**. The frequencies of unaligned chromosomes and multi-polar cells were quantified in control and Incenp-overexpressing (Incenp-OE) cells derived from Tp53-WT and Tp53-KO MEF; n=3. Scale bar, 5.8 µm (A.) and 11.6 µm (D). Statistical analysis was performed using a two-tailed paired Student t-test (B and E); n. s. not significant, *P <0.05, **P <0.01, *** P <0.001, **** P <0.0001.

Fig. S6

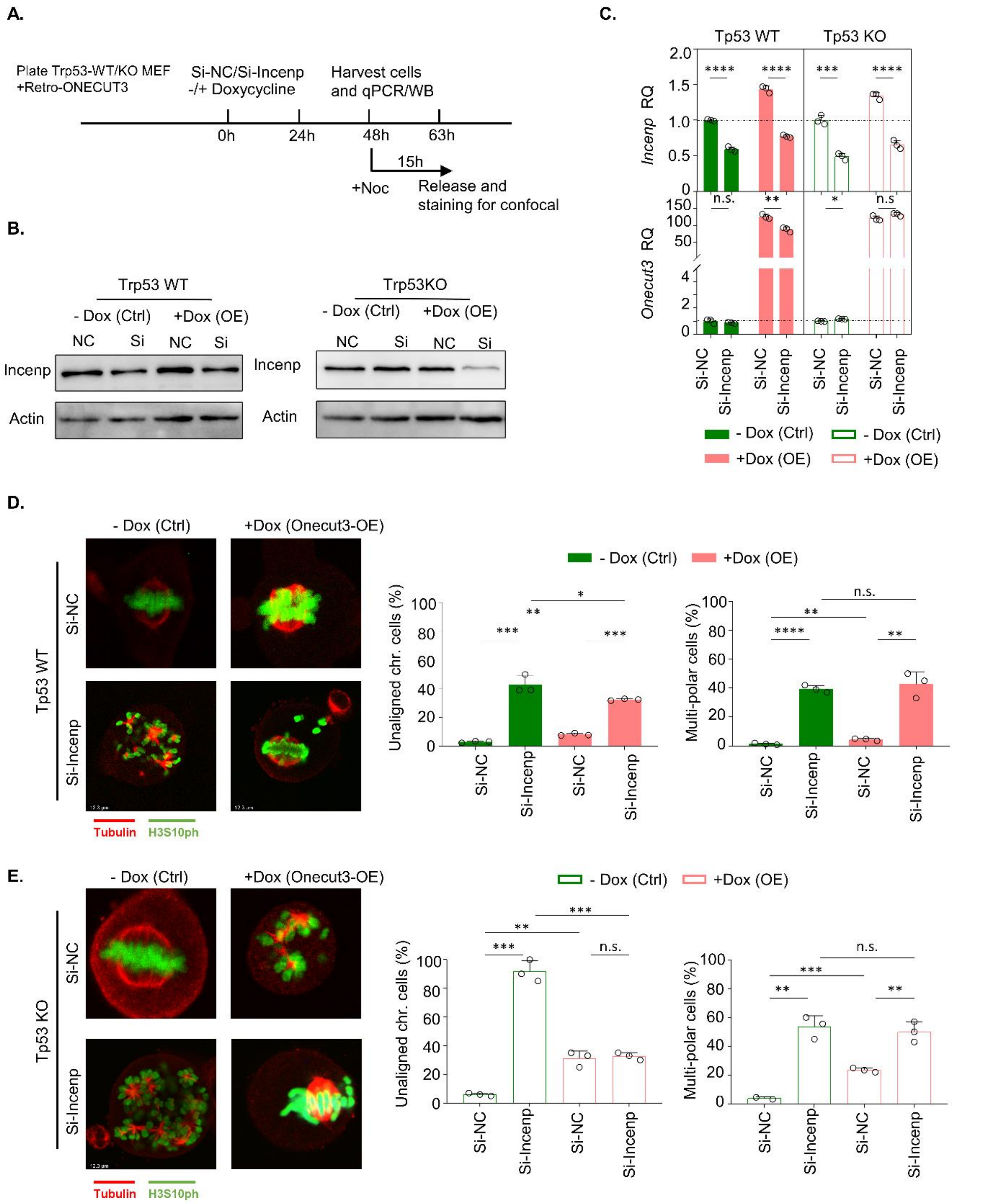

Supplemental Figure 6 (Related to Figure 4). Overexpression of ONECUT3 leads to dysregulation of Chromosomal Passenger Complex and mitotic defects.

A. Schematic overview of the experiment: in the setting of Tp53-WT or Tp53-KO MEF cells that were stably transduced with Retro-ONECUT3, the reduction of Incenp expression was achieved

using small interfering RNA (siRNA), and this reduction was coordinated with the regulation of Onecut3 expression, both in the absence and presence of doxycycline (DOX). After a period of 48 hours, the cells were collected for quantitative polymerase chain reaction (qPCR) or Western blotting (WB). Additionally, to ensure synchronization of the cell cycle, nocodazole was introduced for an additional 15 hours, followed by washout for immuno-staining and confocal analysis. **B-C**. The cell lysates were harvested after the treatment with or without 48h Doxycycline (100 ng ml−1) in control or Incenp-silenced Tp53-WT and Tp53-KO MEF and were then blotted against anti-Incenp antibodies (B.) or quantitated of mRNA level of *Incenp* and *Onecut3* (C.). Loading control is *Actin*. **D**. Left: The representative co-immunostaining image against H3S10ph (green) and Tubulin (red) of control or Incenp-silenced Tp53-WT MEF with (red box) or without Onecut3-OE (green box); Right: The frequencies of unaligned chromosomes and multi-polar cells were quantified in control (Si-NC) or si-Incenp cells; n=3. **E.** Corresponding Tp53-KO part as D**.** Scale bar, 12.3 μm (D-E.); Statistical analysis was performed using a two-tailed paired Student t-test (B and E); n. s. not significant, *P <0.05, **P <0.01, *** P <0.001, **** P <0.0001.

Fig. S7

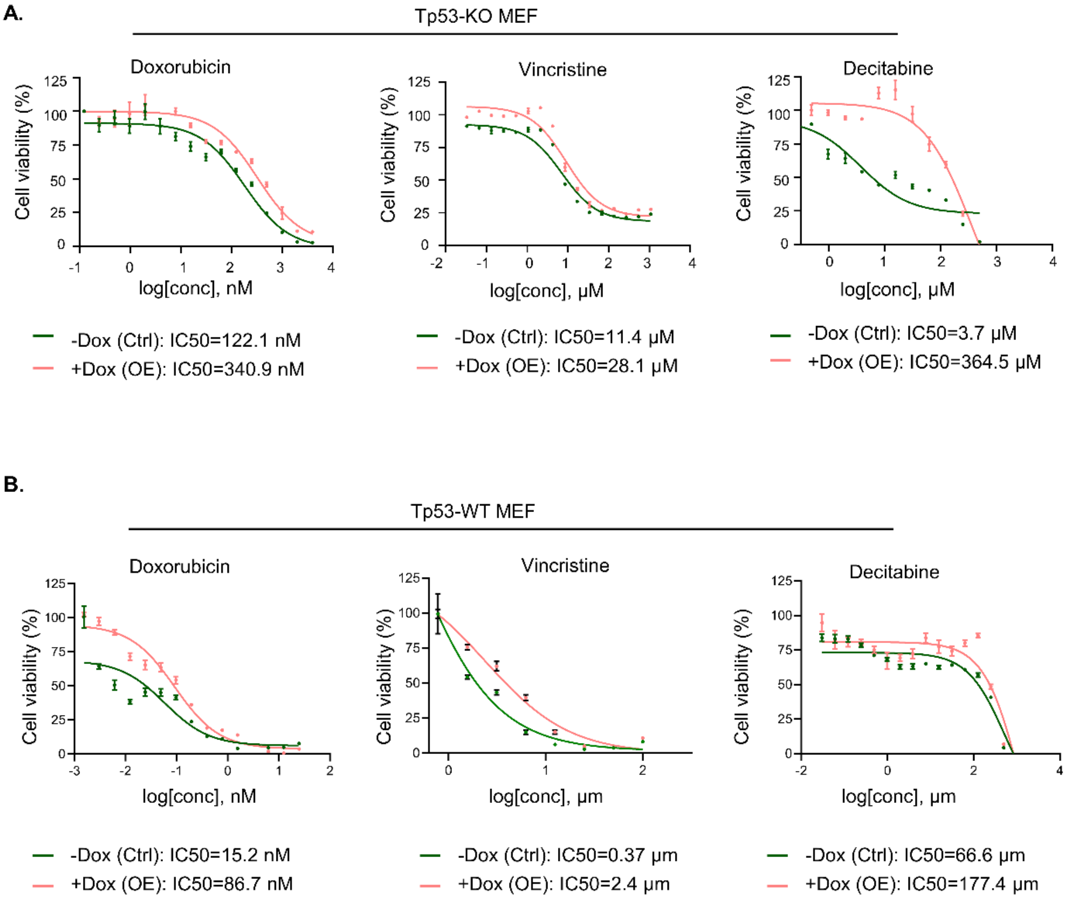

Supplemental Figure 7 (Related to Figure 5) ONECUT3-overexpressing cells present multiple drug resistance, which could be mitigated by targeting to ONECUT3-CPC axis.

A-B. Overexpressed Onecut3 in Tp53-KO MEFs were induced for 7-10 days, and the control cells (-Dox) were cultured simultaneously. The cells were later treated with the chemo-drugs for 48 hours, and the cell viability was tested by CellTiter-Luminescence. The drug sensitivity of the topoisomerase II inhibitor, Doxorubicin (left), the tubulin inhibitor Vincristine (middle), and the DNA methyltransferase inhibitor Decitabine (right) was indicated as IC50. **B.** Overexpressed Onecut3 in Tp53-WT MEFs was also induced in the same condition as above and treated with Doxorubicin (left), Vincristine (middle), and Decitabine (right).

Fig. S8

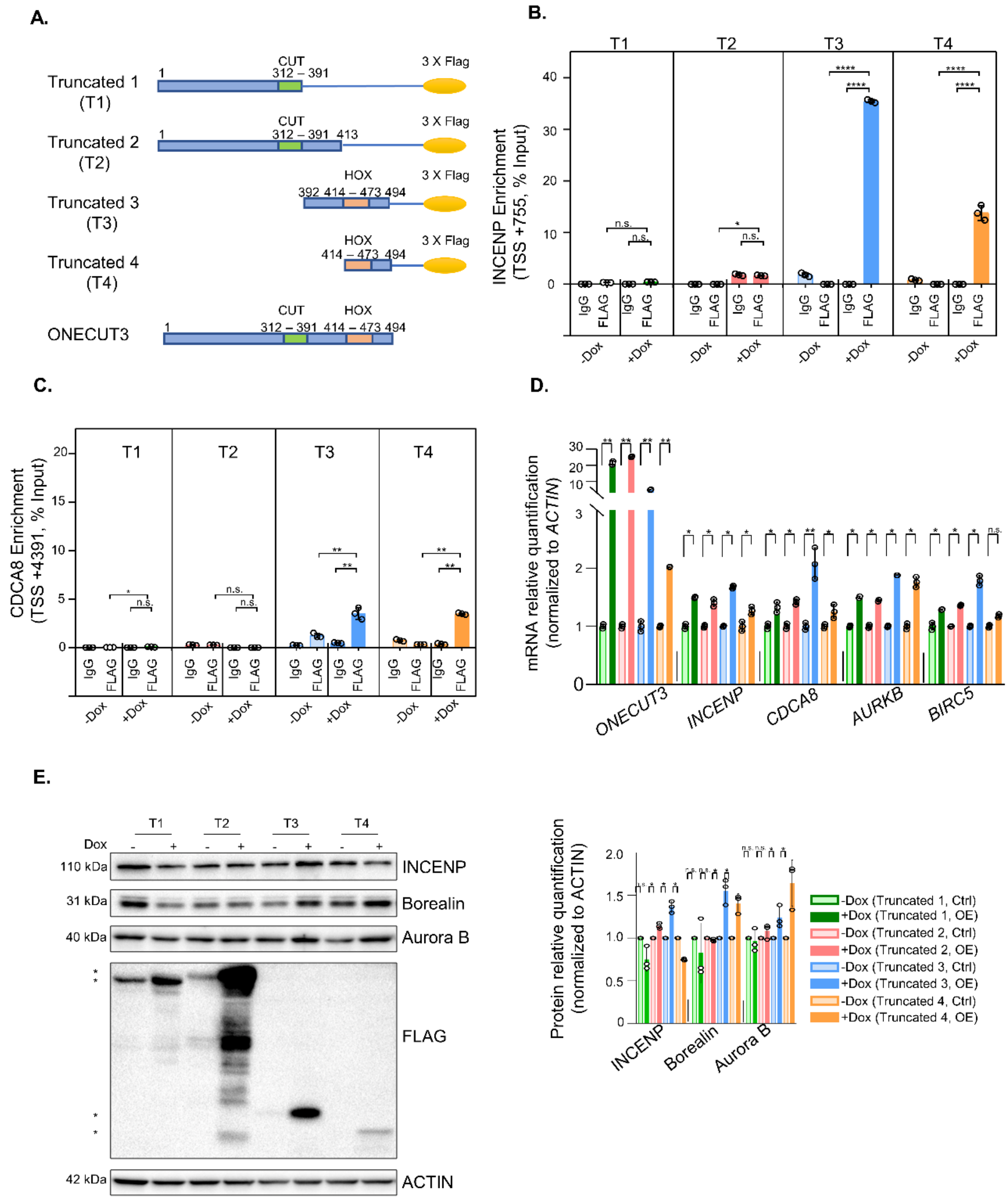

Supplemental Figure 8 (Related to Figure 5) ONECUT3-overexpressing cells present multiple drug resistance, which could be mitigated by targeting to ONECUT3-CPC axis.

A. Schematic representation of ONECUT3 protein. The full length of ONECUT3 protein includes two domains: CUT (green) and HOX (Homeodomain, orange). The plasmids of truncated ONECUT3 with 3x Flag-tag were constructed. **B-C.** Truncated variants of FLAG-tagged ONECUT3 plasmids were transiently transfected in HEK293T cells. ChIP-qPCR assays were

conducted using IgG and FLAG antibodies after 48 hours of Doxycycline induction. The graph shows the comparative analysis o of INCENP (B) and CDCA8 (C) enrichment in control (-Dox) and truncated-ONECUT3-OE (+Dox); n=3. **D-E.** qPCR and Western-blot were also conducted to assess the mRNA (D) and protein levels of CPC components (E).

Fig. S9

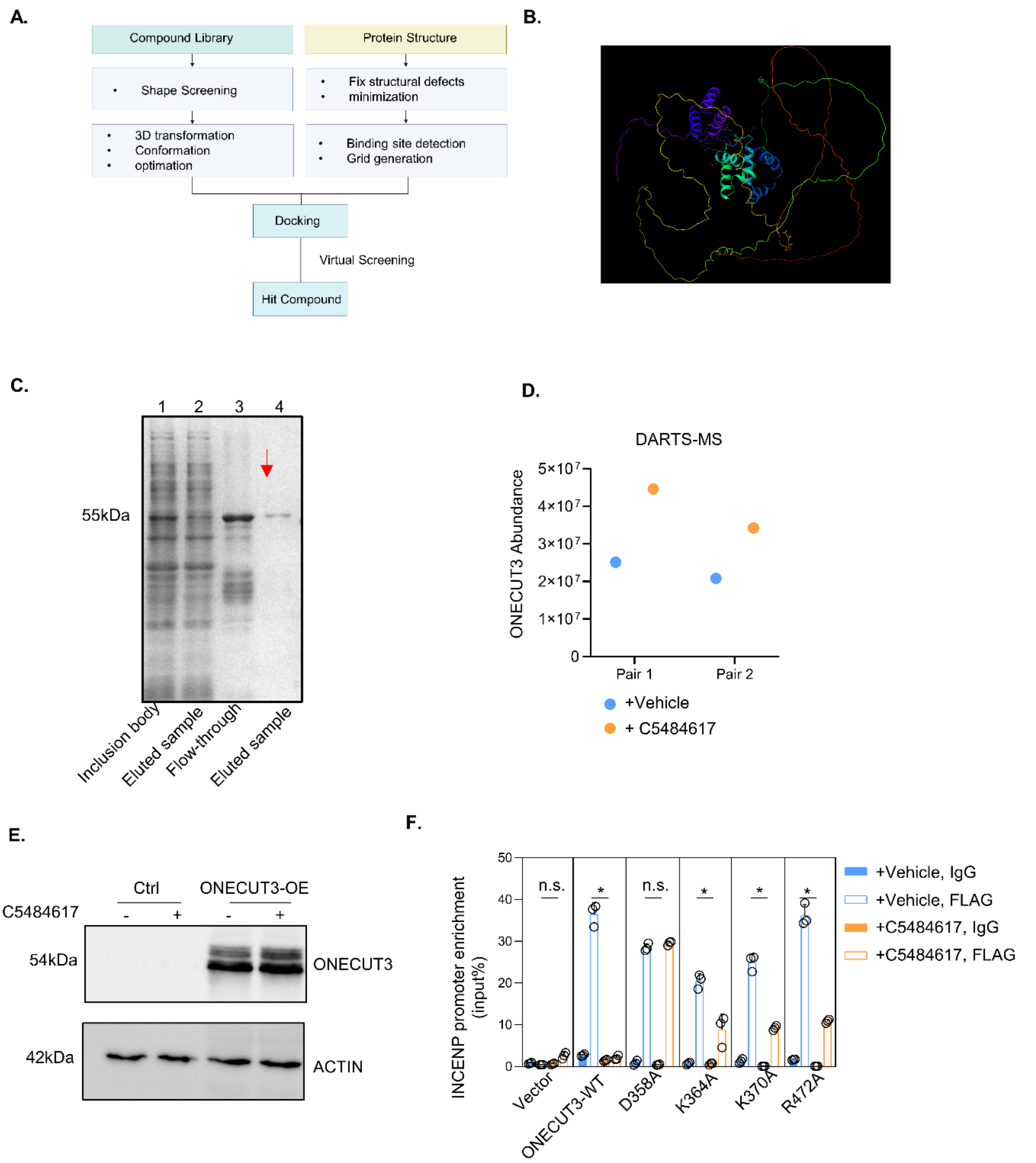

Supplemental Figure 9 (Related to Figure 5) ONECUT3-overexpressing cells present

multiple drug resistance, which could be mitigated by targeting to ONECUT3-CPC axis.

A. Virtual Flow for Virtual Screening. Alpha-Fold2 was used to obtain the three-dimensional structure of the human ONECUT3 protein, and the hydrophobic core cavity near D358, K364, R468, N471, and R472 was served as a possible binding site. As the target region, hydrogen bonds, salt bridges, etc., were added, which were used as structural templates for subsequent virtual screening. Subsequently, we used the 500,000 compounds from Hit2Lead compound library (ChemBridge) for 3D optimization. **B.** The three-dimensional structure of the human ONECUT3 protein was modeled using the Alpha-Fold v2.0. **C**. Western blot analysis for recombinant ONECUT3 protein purified the inclusion body proteins using Ni-Smart affinity chromatography. Lane 1: inclusion body; lane 2: flow-through; lane 3-4: eluted sample; **D**. The target hits were obtained by LC–MS/MS data after Drug Affinity Responsive Target Stability (DARTS); n =2. **E**. Western blot analysis of ONECUT3 in ONECUT3-OE HEK293T cells treated with C5484617 for 48 hours; **F**. HEK293T cells were transiently transfected with control (MSCV-vector), ONECUT3-OE (MSCV-ONECUT3-WT) and ONECUT3 point mutation (ONECUT3-D358A, ONECUT3-K364A, ONECUT3-K370A, and ONECUT3-R472A) constructs for a duration of 48 hours. Following this, ChIP-qPCR assays were performed using IgG and FLAG antibodies. after an additional 36-hour treatment with either Vehicle (DMSO) or 2.5 μM C5484617. The graph illustrates the comparative analysis of the enrichment of INCENP promoter in different treatment groups; N=3.

Fig. S10

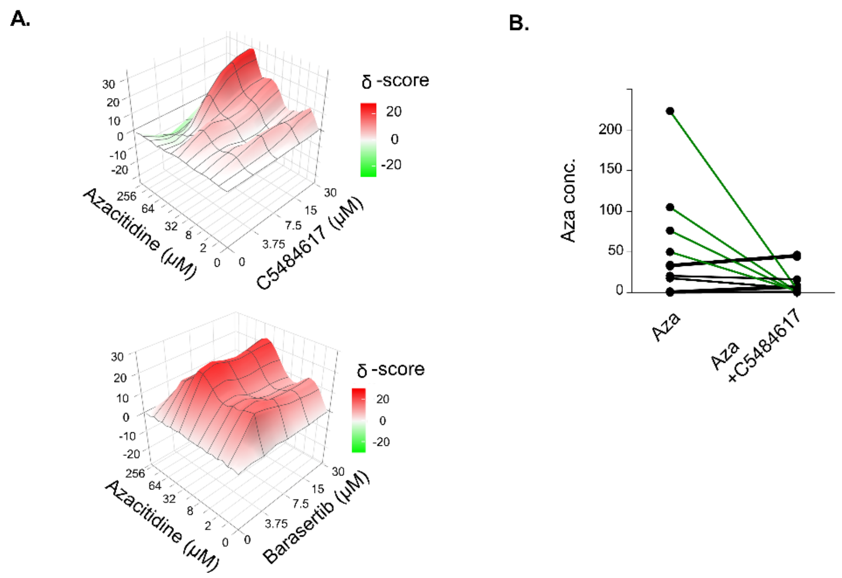

Supplemental Figure 10 (Related to Figure 5) ONECUT3-overexpressing cells present multiple drug resistance, which could be mitigated by targeting to ONECUT3-CPC axis.

A. The Overexpressed Onecut3 in Tp53-KO MEFs was induced for 7 days, and the cells were later treated with the above drugs for 48 h. Dose-response surface for Azacitidine plus C5484617 (lead compound targeting ONECUT3) and Azacitidine plus Barasertib was calculated by SynergyFinder 2.0. ZIP synergy score of Azacitidine plus C5484617: 11.925**,** ZIP synergy score of Azacitidine plus Barasertib: 15.276. **B.** The IC50 of Azacitidine in ‘Azacitidine alone’ group, in ‘Azacitidine with 1μM C5484617’ group; the green line indicates that the concentration of Azacitidine decreased after the combination with C5484617’.

Fig. S11

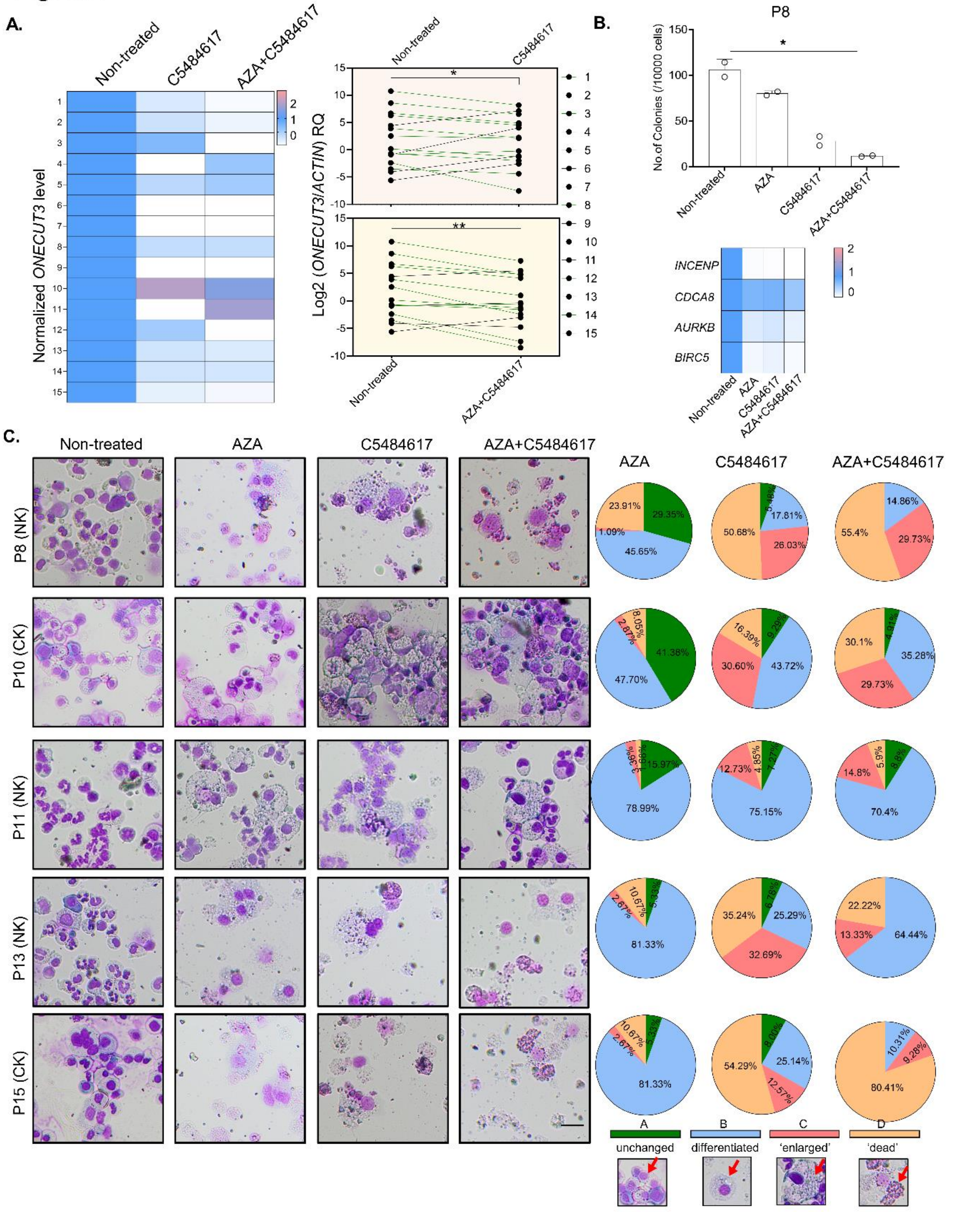

Supplemental Figure 11 (Related to Figure 5) ONECUT3-overexpressing cells present multiple drug resistance, which could be mitigated by targeting to ONECUT3-CPC axis.

A. Left: The mRNA level heatmap of *ONECUT3* was normalized in 15 newly diagnosed MDS patients before and after treatment with C5484617 and Azacitidine with C5484617. Right: Each

data point represents the individual expression of *ONECUT3* before and after treatment with C5484617 and Azacitidine with C5484617; the green line indicates that the level of *ONECUT3* decreased after treatment. **B.** The representative data from the BM mononuclear cells of MDS patient following 48 hours of drug treatments (Azacitidine, C5484617, Azacitidine with C5484617): the quantification of clone numbers (upper), and the assessment of *ONECUT3*/*INCENP*/*CDCA8*/*AURKB*/*BIRC5* mRNA levels (lower). **C.** The representative images (left) and proportions (upper right) of morphological changes in five patients diagnosed with MDS following treatment with Azacitidine, C5484617, Azacitidine with C5484617 for 48 h; a schematic diagram illustrating the morphological changes associated with types A-D (lower right). Scale bar: 100 μm.

Supplemental Table 1. Clinical characteristics of 5 paired patients

No.	Gender	Age	Blast (%)	Karyotype	Mutation
# CK1	Female	60	5	46, XX, t (1;21)(p11;q22), del(5)(q31), del(20)(q11)[1]/46, idem,? Inv(2)(p11q37)[8]/46, XX[1].	*NRAS*, *JAK2*
# NK1	Female	69	6.5	46, XX[20].	*JAK2*
# CK2	Male	66	11	39, XY, -7,-12,-13, der (14;15)(q10;q10), -17,-18,-19,-20,+mar[3]/39, idem, +mar [1].	*TET2*
# NK2	Male	77	14	46, XY[20].	*EZH2*, *ASXL1*, *RUNX1*
# CK3	Male	74	3	43, XY, der (5)(q15), -7,-8,dic (12)(p10)[5]/46, XY [5].	*U2AF1*
# NK3	Male	77	6	46, XY[20].	*U2AF1*
# CK4	Male	33	6	40-42, XY, -4[5], del(5)(q31), -7, -10[8], -18, -19[3],-21*2, -22,+1~2mar, 1 lace [6][10].	*CBL*, *BCOR*, *PTPN11*, *JAK2*
# NK4	Male	23	4.5	46, XY[20].	*BCOR*, *JAK2*
# CK5	Female	45	2	51-52, XX, +1,+2, +4, +5, del (5)(q22), -7[2],-9[2],-13[3],+15[4],+19[3],+21[3],+1-3 mar, 1min [3][5].	*PTPN11*, *ZRSR2*
# NK5	Female	51	3	46, XX[20].	*ZRSR2*

Supplemental Table 2: The list of top 100-upregulated genes by the differential analysis from the paired RNA-seq (CK vs. NK)

Gene name	log2FoldChange	p-value	Gene name	log2FoldChange	p-value
PAPPA-AS1	-8.370247706	1.55E-06	*ZNF385D-AS2*	-5.1811	0.01574
MIR3945HG	-7.568413225	8.59E-06	*ZBTB8OS*	-5.1721	0.01655
CLDN22	-7.25187269	9.84E-05	*TDRP*	-5.1954	0.01762
Ac100800.1	-5.26128681	0.00029	*C7orf61*	-5.1265	0.01768
INHBA-AS1	-6.23209857	0.00031	*LINC01767*	-5.1647	0.01803
AC004817.4	-6.612589558	0.00033	*AC010542.3*	-5.1364	0.01897
GPR32	-6.938259791	0.00036	*TMEM185B*	-5.2616	0.01899
RPL23AP40	-5.618926686	0.0005	*CSGALNACT1*	-5.2052	0.01908
SDCBPP1	-4.381426879	0.00075	*AC131280.1*	-5.2174	0.01956
GPR171	-3.023445181	0.00079	*MTATP6P29*	-5.1133	0.01962
AL138787.1	-6.385364224	0.00082	*CYBB*	-4.746	0.02051
ATP5F1E	-4.232666215	0.00098	*MIR4252*	-5.1424	0.02137
PIWIL4	-6.329327459	0.00104	*AC090970.3*	-5.0423	0.02225
OPTN	-6.051879115	0.00122	*AC092608.1*	-5.0166	0.02285
AL158840.1	-5.810897049	0.0013	*PSMA2P1*	-5.1562	0.02326
SRGAP3-AS3	-6.062945429	0.00172	*RPL17P40*	-5.1196	0.02477
AC007919.1	-5.958300294	0.002	*MTCO1P29*	-5.0638	0.02541
MGC15885	-5.574965521	0.00247	*ZNF81*	-5.0609	0.02647
ONECUT3	-5.777742851	0.00254	*AL591770.1*	-4.9637	0.02747
AC110603.1	-5.946799551	0.00257	*RNA5SP39*	-4.9922	0.02942
RIOK2	-5.85588304	0.00259	*P2RX7*	-5.0198	0.02958
AL669841.1	-5.951848554	0.00298	*RNA5SP187*	-4.9039	0.02964
AC090260.1	-6.132449441	0.00298	*AL662844.4*	-4.9126	0.02998
BNIP3P16	-5.676970491	0.0039	*AP003086.1*	-4.9234	0.0307
C15orf56	-5.792683118	0.00488	*TTC30A*	-4.9299	0.0316
DNAAF3	-5.897917587	0.00513	*Y_RNA*	-4.8798	0.03193
HCG27	-5.663778551	0.0053	*AC008806.1*	-4.9484	0.03195
RNY1P15	-5.63205437	0.0053	*AL928654.2*	-4.9736	0.03303
EREG	-5.804715794	0.0053	*KLF2*	-4.9964	0.03335
NME7	-5.007978454	0.00552	*ARL4AP3*	-4.9798	0.03435
AC110079.2	-5.90122472	0.00597	*FGF11*	-4.9364	0.03445
FPR3	-4.869901424	0.00633	*TRPM7*	-4.9082	0.03574
AP006216.1	-5.509571023	0.00637	*LINC02022*	-4.8374	0.03603
AL035661.1	-4.97564528	0.0071	*ZNF766*	-4.8442	0.03616
FFAR2	-5.596150296	0.00763	*RPSAP51*	-4.8144	0.03699
RNU6-1257P	-5.073178536	0.00787	*AC097639.2*	-4.7981	0.03782
RNU6-733P	-5.508352323	0.00858	*MS4A8*	-4.884	0.03784

CTHRC1	-4.982253764	0.00919	*ITGA2*	-4.8572	0.03806
CELF2	-4.97469858	0.0094	*AC141002.1*	-4.8572	0.03806
BNIP3P38	-5.405390269	0.00973	*NGRN*	-4.7819	0.0398
AC008758.2	-5.471251242	0.00988	*UCA1*	-4.7866	0.04
BCL2A1	-5.462762914	0.01049	*AC007950.2*	-4.7866	0.04
TGFB3-AS1	-5.357497583	0.01058	*RPL12P32*	-4.8008	0.04013
AL451074.1	-4.874554132	0.01104	*BNIP3P26*	-4.8173	0.04066
DQX1	-5.361344201	0.01126	*SAG*	-4.7985	0.04093
RBBP4	-4.877885709	0.01129	*TCFL5*	-4.8081	0.04278
ROPN1L-AS1	-5.015593848	0.01213	*AC133528.1*	-4.7533	0.04437
LINC01864	-4.919622491	0.01246	*AC020611.2*	-4.8273	0.04497
AL139082.1	-5.260029401	0.01288	*AL500527.2*	-4.8283	0.04512
AC104090.1	-5.403072088	0.01321	*AL031848.1*	-5.5109	0.05107

Supplemental Table 3: Univariable and multivariable analysis of factors associated with overall survival in MDS patients

Variables	HR (95% CI)	Univariate analysis P value*	HR (95% CI)	Multivariate analysis P value*
Age (>60yrs VS <60yrs)	1.26 (0.69-2.31)	0.46	1.26 (0.69-2.30)	0.460
Gender (Male/female)	1.46 (0.78-2.73)	0.24	1.64 (0.78-2.73)	0.109
IPSS-R Higher-risk (Higher VS Lower)	4.67 (2.18-9.98)	0.001*	3.98 (1.92-8.24)	0.001*
Karyotype group (Normal VS Complex & Abnormal)	0.79 (0.51-1.24)	0.311	0.79 (0.50-1.23)	0.291
Log *ONECUT3* RQ (High/Low)	2.30 (1.20-4.42)	0.012*	2.00 (1.06-3.78)	0.033*

Supplemental Table 4. Clinical characteristics of 165 MDS patients with TP53 Wild type

	Total (n=165)	Low *ONECUT3* expression (n=56)	High *ONECUT3* expression (n=109)	P value*
Age, median (range)	57 (15-81)	57.5 (17-77)	56.0 (15-81)	0.586
Gender (male/female)	1.6 (101/64)	1.5 (34/22)	1.6 (100/63)	0.933
WBC (*109/L), median (range)	2.6 (0.6-9.3)	2.9 (0.7-9.3)	2.6 (0.6-8.96)	0.211
ANC (*109/L), median (range)	1.2 (0.1-8.2)	1.4 (0.2-5.4)	1.2 (0.1-8.2)	0.221
Hemoglobin (g/dL), median (range)	80 (38-144)	81.5 (38.0-138.0)	80.0(39.0-144.0)	0.895
Platelets (*109/L), median (range)	57 (3-534)	58.5 (8.0-439.0)	55.0 (3.0-534.0)	0.652
BM blasts (%), median (range)	3.0 (0-19)	3.0 (0-18.0)	3.0 (0-19.0)	0.446
2016 WHO subtypes, n, (%)				0.083
MDS-U	12 (7.3%)	8 (14.3%)	4 (3.6%)	
SLD	16 (9.7%)	4 (7.1%)	12 (10.9%)	
MLD	61 (37.0%)	20 (35.7%)	41 (37.3%)	
RS	13 (7.9%)	6 (10.7%)	7 (6.4 %)	
EB-1	34 (20.6%)	12 (21.4%)	21(19.1%)	
EB-2	28 (17%)	5 (8.9%)	23 (22.9%)	
5q- syndrome	1 (0.6%)	0 (0%)	1 (1.2%)	
IPSS-R risk categories, n, (%)				0.094
Very good	7 (4.2%)	2 (3.6%)	5 (4.6%)	
Good	41 (24.7%)	15 (26.8%)	26 (23.9%)	
Intermediate	50 (30.1%)	21 (37.5%)	29 (26.6%)	
Poor	46 (27.7%)	16 (28.6%)	30 (27.5%)	
Very poor	21 (12.7%)	2 (3.6%)	19 (17.4%)	
Karyotype subgroup1				0.009*
Normal	93 (56.0%)	38 (67.9%)	55 (50.5%)	
1-2 Abnormal	60 (36.1%)	18 (32.1%)	42 (38.5%)	
Complex (≥3)	12 (7.2%)	0 (0%)	12 (11%)	
Karyotype subgroup 2				0.079
Normal	93 (56%)	38 (67.9%)	55 (50.5%)	
Numerical abnormal	23 (13.9%)	8 (14.3%)	15 (13.8%)	
Structural abnormal	32 (19.3%)	8 (14.3%)	24 (22%)	
Numerical and structural abnormal	17 (10.2%)	2 (3.6%)	15 (13.8%)	

Supplemental Table 5. The correlation of *ONECUT3* mRNA expression and recurrent gene mutation in MDS patients

Mutational variable	Mutation status	N	Low *ONECUT3* expression (n=56)	High *ONECUT3* expression (n=109)	P value*
ASXL1	WT[+]	140	48	92	0.824
	Mutated	25	8	17	
BCOR	WT	151	53	98	0.386
	Mutated	14	3	11	
CBL	WT	157	53	104	0.999
	Mutated	8	3	5	
CEBPA	WT	160	55	105	0.663
	Mutated	5	1	4	
CREBBP	WT	157	53	103	0.719
	Mutated	8	2	6	
CSF3R	WT	160	53	107	0.338
	Mutated	5	3	2	
CUX1	WT	161	55	106	0.999
	Mutated	4	1	3	
DNMT3A	WT	151	51	100	0.999
	Mutated	14	5	9	
EP300	WT	157	53	104	0.999
	Mutated	8	3	5	
ETV6	WT	157	52	105	0.446
	Mutated	8	4	4	
EZH2	WT	157	52	105	0.446
	Mutated	8	4	4	
FLT3	WT	160	55	105	0.663
	Mutated	5	1	4	
GATA1	WT	164	56	108	0.999
	Mutated	1	0	1	
GATA2	WT	163	55	108	0.999
	Mutated	2	1	1	
IDH1	WT	158	56	102	0.097
	Mutated	7	0	7	
IDH2	WT	159	51	108	0.018*
	Mutated	6	5	1	
JAK2	WT	161	53	108	0.114
	Mutated	4	3	1	
KIT	WT	163	55	108	0.999

Continued Supplemental Table 5. The correlation of *ONECUT3* mRNA expression and recurrent gene mutation in MDS patients

Mutational variable	Mutation status	N	Low *ONECUT3* expression (n=56)	High *ONECUT3* expression (n=109)	P value*
	Mutated	2	1	1	
KMT2D	WT	156	50	106	0.033*
	Mutated	9	6	3	
KRAS	WT	159	56	103	0.097
	Mutated	6	0	6	
NPM1	WT	163	56	107	0.549
	Mutated	2	0	2	
NRAS	WT	157	53	104	0.999
	Mutated	8	3	5	
PHF6	WT	156	56	100	0.029*
	Mutated	9	0	9	
PTPN11	WT	158	55	103	0.425
	Mutated	7	1	6	
RUNX1	WT	141	49	92	0.382
	Mutated	24	7	17	
SETBP1	WT	157	53	102	0.999
	Mutated	10	3	7	
SF3B1	WT	147	47	100	0.185
	Mutated	18	9	9	
SRSF2	WT	159	53	106	0.409
	Mutated	6	3	3	
TET2	WT	139	49	90	0.502
	Mutated	26	7	19	
TP53	WT	165	56	109	-
	Mutated	-	-	-	
U2AF1	WT	140	51	89	0.168
	Mutated	25	5	20	
WT1	WT	159	55	104	0.665
	Mutated	6	1	5	
ZRSR2	WT	159	54	105	0.999
	Mutated	6	2	4	

+WT, wide type, MT, mutant type; * P-value is less than 0.05.

Supplemental Table 6 Table of TOP20 Hit compounds from the virtual screening.

ChemBridge ID	Docking score	Glide gscore	Glide energy
7889067	-7.336	-7.351	-38.107
5135322	-7.079	-7.141	-31.245
5140914	-6.736	-6.736	-29.155
5264732	-6.724	-6.727	-35.259
7962588	-6.712	-6.727	-38.058
7962588	-6.695	-6.71	-36.33
5135483	-6.578	-6.578	-35.554
5226879	-6.568	-6.571	-32.522
5160423	-6.52	-6.521	-36.273
5160424	-6.518	-6.519	-36.138
5267209	-6.485	-6.508	-41.901
5266986	-6.484	-6.51	-41.387
5989208	-6.473	-6.584	-31.506
5484617	-6.371	-6.556	-39.848
5631271	-6.307	-6.33	-38.377
5405740	-6.257	-6.396	-30.623
5135482	-6.251	-6.252	-30.866
7875890	-6.24	-6.25	-40.685
5160143	-6.239	-6.241	-34.808
6665804	-6.221	-6.221	-32.683

Supplemental Table 7. Table of the predicted proteins of in Liquid Chromatography with tandem mass spectrometry (LC–MS/MS) screening after DARTS.

P-value	Protein
P<0.001	PRRC2C/TXN2/NDRG1/RALA/BICD2/MRPL50/SLC38A9/NOL10/USP19/SUCLG2/CMSS1/RAB17/MTFP1/SLC25A10/COL6A1/ENDOD1/RANBP1/SFSWAP/PRRC2A/MRPS34/RPS29/PDCD2/SPNS1/ACTL7A/GPX1/KLC2/ABAT/RAB5B/ARPC5/GSN/SENP3/MORF4L2/KIF5B/CLCN4/MICOS13/PTPN9/F11R/HYPK/NSMCE3/CTSA/RPS23/VPS51/ATM/GPR89B/ARF5/HBA1/TACC3/ESD/USP48/RAB5A/SLC25A19/CMTM6/MRPL46/TCP11L2/COPG2/DMD/DR1/ANK/IKBKG/ATP5F1D/SCD5/RPL28/NHP2/SLC25A11/VBP1/DSP/EIF4E2/TDRKH/CCDC22/RPL30/STARD3NL/RAB4A/SCN7A/RPS16/PRPS1/SNTB2/CALM3/RPL32/ACOT9/PRDX4/SUOX/RPL36A/RPS26/DNPEP/PPP2R5D/NIPSNAP3A/QPCTL/MBLAC2/LSM3/KRTCAP2/KIAA2013/H2BC18/RPS20/MRPL21/RPL37A/TRIM27/BOLA2/GTPBP1/COX5A/FIS1/RPS11/SERBP1/ARAP1/ZDHHC5/RPL35/RPL26L1/HSPA13/MXRA7/ZC3H7A/AP1B1/NCAPH/HSPA14/MTA1/DNAJC8/TPM4/SLC1A5/RPLP2/RPL34/SMARCE1/MRPS16/SNRPB/RPL22L1/TMEM97/RPAP3/RRM2/XPOT/PAM16/H4C1/MAGT1/RPS25/RIDA/HSPE1/NDUFB7/SH3BGRL/ISOC1/TBRG4/SLC4A1AP/NPC2/CNDP2/JAM3/NTPCR/MRPS17/GET3/CSTB/TLN1/NDUFA13/MRPL3/PRKAA2/LAGE3/MRPS31/TXNDC17/MACROD1/DBT/L1RE1/SNRPA1/POLR3F/APOO/ELOVL/FUCA1/ERG28

	/TMEM65/RPL7A/USP14/ROBO1/CDK7/RCOR1/PURA/MTATP6/CYP2R1/CPSF7/CYCS/MRPL49/AVEN/MYDGF/AP2A1/AKAP17A/ACOT8/RPS13/PCID2/RPS19/DDX46/TMED3/TTC26/RPS15A/GGH/VAPA/CHTOP/GAR1/UBA52/RPL35A/ERH/RPS14/HSD17B11/CDKN2A/RPL27/ENOPH1/CHPT1/PIN4/ABCB1/RPP30/HSPA2/DNAJA2/PML/LSM1/ERC1/ABCB7/RAP1GDS1/PTDSS1/RFC5/RPS28/TSPAN6/PKN2/ERCC3/MAU2/XP32/RPL24/ACADS/BTF3/AIMP1/TOMM22/MRPS14/PFDN2/SYNCRIP/TBCD/RALY/TUBG1/AP2M1/CIAPIN1/HNRNPH3/CAPNS1/WDR45B/TMEM205/FABP5/PSMA3/MCCC1/C1QBP/SNRPD3/EIF2B3/RRP1B/COMP/NOB1/COTL1/MRPL38/PSMA1/SOD1/USP46/ZNF207/APP/ZCCHC8/MIS12/BAG2/STX4/PLEC/ETNPPL/ABCF1/SGSH/UTP20/FMR1/ODR4/DYNC1I2/PIGT/SMCHD1/TIMM13/DHX15/POLR3A/HLADRB1/NPM3/COX4I1/EIF5A/ATPAF1/MAPK3/RPL31/TIMM44/RPL23/MYH6/ANO6/PSEN2/OR1M1/PEF1
P<0.01	FAM186B/TTLL12/RPIA/FANCI/PSMC5/SLC6A9/CLPTM1L/LGALS3BP/STIP1/UBE2L3/CORO1B/CCDC93/ARPC4/SMARCC1/MYL12B/BCS1L/RPS10/CAP1/ACSF2/CFL1/XPNPEP1/PPP4C/OTULINL/NPM1/RPL36/SEC61B/SNRPA/DNAH9/NBAS/SMS/UGP2/SEC11A/MTOR/SURF6/SPCS1/DHX29/RPS18/PARG/SRP19/KHDRBS1/LARP1/UBQLN2/ERCC6L/ATP6V1D/PPIA/MRPL17/HDDC2/TWF1/SLC35F6/MC2R/RPL12/ERP44/RAB10/PFN1/EXOSC8/STRN3/RPL27A/HDAC2/ANKRD13A/RPS17/RPUSD3/BST2/TMX4/ORMDL3/DHX37/RPL23A/PSMB2/SMPDL3B/EMC7/DUT/NLE1/TMEM177/TRMT1/UBE2V1/CD59/SNU13/ADPGK/TRMT5/SPRYD7/SLC25A21/NDUFAF7/PARS2/PSMG1/TFRC/GTF3C5/GPAT4/LARS1/MAP1A/DNAAF5/EXOSC6/HTT/CTR9/SHPK/CISD2/DPM1/CKMT1A/HNRNPDL/TFG/EIF5B/SHOC2/RPL26/NDUFA5/TMPO/LRRC8A/BZW2/NDUFS7/CDKAL1/GCAT/LIPT2/YWHAB/DDX56/TPMT/PPIL4/STUB1/BCKDHA/ERO1B/COPE/TOMM20/MTCH1/ALYREF/SCLY/SRPRB/CHUK/ZW10/EEF1E1/CDK5/USP10/SMARCA4/SCFD2/UCHL1/C8orf33/SPAG9/RMND1/PAK4/PABPN1/H33A/MOGS/THOC5/ANAPC4/PTK2/RPL11/TMLHE/RPL4/MMTAG2/RPL13/IDH3B/EEF1A2/AP3B1/TXN/GAPVD1/ACAA2/RNGTT/MTX1/SLC35A4/PSME3/TACO1/JAGN1/SNX4/GTPBP4/HINT2/SRI/ELP3/RRP1/UBE2N/MAGOH/GET1/HDGFL2/PIR/AKAP8L/PFN2/UBE2O/NUTF2/EPB41L5/PAXBP1/MYL6/USP24/PLAA/TOP2B/THOC2/TRAPPC3/TIMM22/ZC3H18/FDXR/ARL1/DHX16/CTCF/RPS24/MRPS23/WFS1/ANP32E/FASTKD5/APEX1/LARP4B/RPS15/DIABLO/CLCN7/MAP7D1/ANKRD29/PDCD6/GLMN/DNPH1/LDHA/SMC4/FERMT2/LAS1L/PRDX6/LAMP2/NUDC/LAMTOR1/NIP7/KDSR/PSMC2/JAK3/LMNA/LMBR1/NCAPD2/RPS12/VPS52/CTBP1/NUCB2/XAB2/PGM2L1/CSNK2A1/PEX11B/GCLM/DHRS7B/ARPC3/PSMD6/ADAR/TBCA/ETFA/PHAX/IDH3G/ACTR1B/AFDN/LMF2/CDH2/YKT6/VARS2/CHD4/NAA50/SEPTIN3/UGDH/CPOX/MRPL28/DDX10/ORC5/DLAT/PHB/MRPS22/TSN/KIFC1/FTL/ENPP4/CTNND1/PSMA5/GTPBP10/RPL22/AFG3L2/MGME1/RPA2/CCAR2/CDC73/OGFR/SLC27A4/MRPS5/SERPINH1/I

	GSF8/POFUT1/CHP1/SNRPB2/SLC25A23/PSMD7/BLMH/SRP14/RBM4/SUPV3L1/BCKDHB/POLR2C/RUVBL1/EXOSC5/CD81/SCG2/ACSF3/CTSC/ELOB/ABRAXAS2/EPB41/SLC25A24/ARF4/HARS1/MICAL1/DNAH11/NUDT21/SSR4/DERL1/GDI2/NAXE/ANXA2/PFKL/PTTG1IP/C7orf50/POLRMT/SLC25A17/POFUT2/LYPLAL1/ESYT2/INTS8/SRPRA/TRIM28/MPI/RPRD1A/MED23/BZW1/MRPL11/EIF3F/HAND2/AGTRAP/NME1/TMCO1/NME2/PITHD1/SARS1/MTDH/MAP1S/TXNL1/ATP5P/RNASET2/COPS5/TCEA1/PEX14/ATL1/GNPDA1/NUP93/TMEM109/AK4/CACYBP/GSTM2/TEX10/ACAT2/DCUN1D5/TMEM33/WLS/PKM/DNAJB11/DDX42/LIG4/HNRNPH1/GINS2/NUP85/UBE2I/CSNK2A2/SLC7A6/DHX36/ABHD10/NOC3L/MRE11/ATAD3B/HEATR1/ECHS1/SSBP1/COQ9/MDH2/IP6K1/SIGMAR1/MCM7/EIF6/PNP/PLCD3/EIF1AX/MRPL23/MRI1/CLN5/CFL2/SPCS3/RAB18/CTSD/VRK3/BCAP29/SF3A3/SURF4/PEBP1/MAPK14/TXNRD2/LYAR/ACIN1/TMEM70/SAAL1/ARHGAP1/DNAJC13/DCP2/SRP72/GID8/APEH/NUDCD1/SNX5/CDK4/AGBL2/GOT2/MAIP1/SLTM/PPIB/ACP1/TBCE/GPAT3/CENPF/GORASP2/TMED10/INTS3/PGM3/NHLRC2/GAK/MRPS25/CAB39/PLS3/PNO1/ANP32A/LPCAT1/HNRNPC/NOL9/MLYCD/GCDH/OAT/NUP54/PDIA6/RBMXL1/DCTPP1/RBMX/ITPR2/WBP2/ATP5MF/PDXP/DSTN/RDH10/IQGAP2/TMED5/DNAJC10/GCC2/RMDN3/RPLP1/RTN1/HSPA9/FAF2/RANBP9/ATP5F1A/RAP2A/GDPD1/PPAN/SLC25A6/GOLGB1/MRPL37/WRNIP1/SFXN4/VIM/CD151/PMPCA/RBFOX2/UBE2D3/COG1/UBE2R2/MYADM/VAC14/SLC9A3R1/PPME1/GALNT1/MCMBP/PTBP1/RSL1D1/SYNE2/CEBPZ/RAB2A/DYNLL1/ABRACL/ASPH/SRRT/RAB9A/SERPINB6/CCDC51/RHOB/PARK7/PSMB6/SLC29A1/EARS2/HSPB1/RBM39/STT3A/ARGLU1/PUF60/PCMT1/ERCC1/HACD3/TMED1/UFC1/NAT10/DNAJB1/CNP/NDUFB10/DGUOK/PODXL/PRDX5/NOC2L/MANF/APOA1/AP2S1/CYC1/SUPT5H/MRPL10/PPIH/**ONECUT3**/DNAAF10/LUC7L3/MAD2L1/GOLT1B/KCTD12/SAE1/TMEM192/AHSA1/RPL38/ARL6IP5/CSTF2/RPL17/RPN1/CD9/GYS1/CLPTM1/TSPO/GTF3C2/RAB12/MYBBP1A/RAB8A/PPOX/RIPK1/PTRH2/CDK11B/CHERP/MRPS26/SNX8/NDUFS2/EEF1B2/OLA1/RBMS1/SEC22B/UBE2S/PDLIM5/PHB2/HEXB/UPF3B/BPHL/HSCB/PAPOLA/U2AF1/TOMM70/PSMC3/HADHB/PRCP/PGAM5/TRIM33/ALDH3A2/DCTN2/ZNF326/TOR1AIP1/POLDIP2/SAP18/ARIH2/SEC24B/FLOT2/RPL6/FAM98B/PPP3CA/NEMF/RAB11B/PELP1/PDPR/DCD/ATP6V1A/ERGIC1/IARS1/RCC1/ATP5F1B/NT5C3A
P<0.05	QPRT/NAA10/RP2/ATP5PB/FAM50A/ARL6IP1/YTHDF2/TPI1/SAMHD1/USP9X/PRC1/TIAL1/TBCB/COPA/GABARAP/OXA1L/AP1M1/UQCRC2/CCT3/DDX31/SMPD4/NUP205/EIF3K/FARS2/ERMP1/CCDC33/RRAGC/RPL39/TBL3/AGK/FBL/SFXN1/SLC12A4/UBLCP1/PSMC4/OSBP/NOP56/MRPS10/UBE2T/SNX17/NOP58/DDX51/ENAH/EXOSC2/MRPL13/STXBP2/EHD4/XPO7/ACP6/LZIC/MBOAT7/H1-3/TNPO1/SLC39A14/POLR2H/EIF4G1/LTA4H/BRI3BP/PI4K2A/THEM6/SNX9/OPA1/HEXA/OGFOD1/GCA/HS2ST1/UBE2M/AK6/SUMO1/CS/GTF2H2/PCK2/FARSA/A

	CP2/ELAVL1/MTCO2/MRPL20/SOAT1/ATP6V1B2/SF3B6/POLR2A/PSME4/AKR1B1/WDR70/U2AF2/ILF2/MRPL18/S100A13/CCNT1/TTC1/ANKRD28/TPR/CENPH/HNRNPU/NAGK/COPS8/TBC1D4/TMF1/ACSL4/DNMT1/CAB39L/GLUD1/SNRPD2/TIMMDC1/HSPBP1/GSS/PPP4R3A/PTP4A1/COPZ1/PHGDH/SLC25A3/SEPTIN7/DHX8/DCXR/EMC3/FUBP3/CCNK/TRMT1L/RAB5C/OXSR1/DHX30/CPSF2/MDH1/CFAP20/MTHFS/LIMS1/WASHC5/AP1S2/RWDD1/GRPEL1/TAMM41/APRT/DYNC1H1/COX15/BTF3L4/CBR1/RNASEH2B

Supplemental Table 8. Antibody information in this study

Antibody	Source	CAT#
FITC anti-human CD2	Biolegend	300206
FITC anti-human CD3	Biolegend	300305
FITC anti-human CD7	Biolegend	343104
FITC anti-human CD10	Biolegend	312207
FITC anti-human CD11b	Biolegend	301329
FITC anti-human CD14	Biolegend	325603
FITC anti-human CD19	Biolegend	302205
FITC anti-human CD235a	Biolegend	349103
PB anti-human CD38	Biolegend	356627
APC anti-human CD34	Biolegend	343509
Anti-ONECUT3	homemade	detailed in Supplemental Method
Anti-INCENP	Abcam	ab12183
Anti-Aurora B	Abcam	ab2254
Anti-Borealin	Santa Cruz	sc-376635
Anti-Borealin/CDCA8	Proteintech	12465-1-AP
Anti-Survivin	Huabio	ET1602-43
Anti-α-Tubulin	Sigma	T6199
Anti-Tubulin	Abcam	ab6161
Anti-Histone H3 (phospho S10)	Abcam	ab5176
Anti-Flag	Sigma	F3165
Anti-Lamin B1	Abcam	Ab160488
Goat Anti-rabbit IgG (H+L), F(ab')2 Fragment, Alexa Fluor 488 Conjugate	Cell Signaling Technology	s4412
Goat anti-Mouse IgG (H+L), TRITC	Invitrogen	A16071
Goat Anti-rat IgG (H+L), F(ab')2 Fragment,	Cell Signaling	s4414

Alexa Fluor 647 Conjugate	Technology

Supplemental Table 9. Chemical reagents in this study

Chemical Reagent	Source	CAT#
Puromycin	InvivioGen	ant-pr-1
Doxycycline	Selleck	S5159
5-fluorouracil (5-FU)	Selleck	S1209
Nocodazole	Selleck	S2775
Barasertib (AZD1152-HQPA)	Selleck	S1147
C5484617	Chembridge	5484617
Azacitidine	Sigma	A2385
Decitabine	Sigma	A3656
Vincristine sulfate	Selleck	S1241
Doxorubicin	Pfizer	NSC-123127

Supplemental Table 10. Sequences of primers used for the TP53 genotype

Name	Sequence
Tp53 Common	TGGATGGTGGTATACTCAGAGC
Tp53 Mutant Forward	CAGCCTCTGTTCCACATACACT
Tp53 Wild type Forward	AGGCTTAGAGGTGCAAGCTG

Supplemental Table 11. Sequences of primers used for the constructs of overexpression plasmids

Name	Sequence
Retro-ONECUT3-Full length-3FLAG Forward	CGGAATTCGCCACCATGGAGCTGAGCC
Retro-ONECUT3-Full length-3FLAG Reverse	TGAGATCTGACGGGCACCGGAGCGATCGC
Retro-ONECUT3-Truncated 1 Forward	TCCTACCCTCGTAAAGAATTCGCCACCATGGAGCT GAGCCTGGAGAGCCTG
Retro-ONECUT3-Truncated 1 Reverse	GAAGGGCCCTCTAGACTCGAGTTCGAACATGCGCT GGAACTCTGGCTC
Retro-ONECUT3-Truncated 2 Forward	TCCTACCCTCGTAAAGAATTCGCCACCATGGAGCT GAGCCTGGAGAGCCTG
Retro-ONECUT3-Truncated 2 Reverse	GAAGGGCCCTCTAGACTCGAGTTCGAACTGCAGG

	GCGCGCTCCTTCTG
Retro-ONECUT3-Truncated 3 Forward	TCCTACCCTCGTAAAGAATTCGCCACCATGTCGGC GCTGCGCTTGGCAGCC
Retro-ONECUT3-Truncated 3 Reverse	GAAGGGCCCTCTAGACTCGAGTTCGAAGGCCTTG GAGAAAGTGGCCGT
Retro-ONECUT3-Truncated 4 Forward	TCCTACCCTCGTAAAGAATTCGCCACCATGCCCAA GAAGCAGCGCCTGGTG
Retro-ONECUT3-Truncated 4 Reverse	GAAGGGCCCTCTAGACTCGAGTTCGAAGGCCTTG GAGAAAGTGGCCGT
MSCV-ONECUT3-D358A Forward 1	CTCCTTCTCTAGGCGCCGGAATTCGCCACCATGGAG CTGAGCCTGGAGAGC
MSCV-ONECUT3-D358A Reverse 1	GGGGTTGCGCAGCAGAGCGGAGAGCGT
MSCV-ONECUT3-D358A Forward 2	CAGGGCACGCTCTCCGCTCTGCTGCGC
MSCV-ONECUT3-D358A Reverse 2	ATCCCGCTCGAGCAATTGGGATCCGGCTGATCAGCG GGTTTAAACTCACTTGTCATCGTCATCCTTGTAG
MSCV-ONECUT3-K364A Forward 1	CTCCTTCTCTAGGCGCCGGAATTCGCCACCATGGAG CTGAGCCTGGAGAGC
MSCV-ONECUT3-K364A Reverse 1	ATTTGAGCTTGCTCCACGGAGCGGGGTTGC
MSCV-ONECUT3-K364A Forward 2	CTGCTGCGCAACCCCGCTCCGTGGAGC
MSCV-ONECUT3-K364A Reverse 2	ATCCCGCTCGAGCAATTGGGATCCGGCTGATCAGCG GGTTTAAACTCACTTGTCATCGTCATCCTTGTAG
MSCV-ONECUT3-K370A Forward 1	CTCCTTCTCTAGGCGCCGGAATTCGCCACCATGGAG CTGAGCCTGGAGAGC
MSCV-ONECUT3-K370A Reverse 1	GGTCTCGCGGCCGGAAGCGAGCTT
MSCV-ONECUT3-K370A Forward 2	CCGTGGAGCAAGCTCGCTTCCGGC
MSCV-ONECUT3-K370A Reverse 2	ATCCCGCTCGAGCAATTGGGATCCGGCTGATCAGCG GGTTTAAACTCACTTGTCATCGTCATCCTTGTAG
MSCV-ONECUT3-K364A Forward 1	CTCCTTCTCTAGGCGCCGGAATTCGCCACCATGGAG CTGAGCCTGGAGAGC
MSCV-ONECUT3-K364A Reverse 1	CTCCTCAGCCCAAGCGTTCATGCAGCG
MSCV-ONECUT3-K364A Forward 2	ATGAACGCTTGGGCTGAGGAGCCC
MSCV-ONECUT3-K364A Reverse 2	ATCCCGCTCGAGCAATTGGGATCCGGCTGATCAGCG GGTTTAAACTCACTTGTCATCGTCATCCTTGTAG

293

294 **Supplemental Table 12. Sequences used for the ONECUT3 genotype in HSPC**

Name	Sequence
ONECUT3-Forward	TGTGAAATTTGTGATGCTATTGCT
ONECUT3-Reverse	AGCGTTTGGCAATGTGCTTT

Supplemental Table 13. Sequences of SiRNA

Name	Sequence
SiRNA-Incenp sense	CUCCUAUCCUGCUGAAUAATT
SiRNA-Incenp antisense	UUAUUCAGCAGGAUAGGAGTT
SiRNA-NC sense	UUCUCCGAACGUGUCACGUTT
SiRNA-NC antisense	ACGUGACACGUUCGGAGAATT

Supplemental Table 14. Sequences of primers used for Real-time PCR

Name	Sequence
Human ACTIN Forward	ACTCTTCCAGCCTTCCTTCC
Human ACTIN Reverse	AGCACTGTGTTGGCGTACAG
Human ONECUT3 Forward	CGGATCCTGTGTCGCTCT
Human ONECUT3 Reverse	CACATCCTGCGGAAGGTCTC
Human INCENP Forward	AAGCTCATGGAGTTTCTCTGC
Human INCENP Reverse	CGTCTCTTCTTCCGTCGGTTC
Human CDCA8 Forward	GCAGGAGAGCGGATTTACAAC
Human CDCA8 Reverse	CTGGGCAATACTGTGCCTCTG
Human AURKB Forward	CACATTTGACGAGCAGCGAAC
Human AURKB Reverse	CACATTGTCTTCCTCCTCAGGG
Human BIRC5 Forward	AGGACCACCGCATCTCTACAT
Human BIRC5 Reverse	AAGTCTGGCTCGTTCTCAGTG
Mouse Actin Forward	TGTACCCAGGCATTGCTGAC
Mouse Actin Reverse	AACGCAGCTCAGTAACAGTCC
Mouse Onecut3 Forward	CGGATCCTGTGTCGCTCT
Mouse Onecut3 Reverse	CACATCCTGCGGAAGGTCTC
Mouse Incenp Forward	GAGGCTGAGCGCATGTTTATC
Mouse Incenp Reverse	CCTCACGGGATCTCTGTTTTC
Mouse Cdca8 Forward	AAAAGCGAAAGGTAATCGAGGT
Mouse Cdca8 Reverse	TGCAGATCGAAGATTCTTATGGC
Mouse AurkB Forward	ATCGGGGTGCTCTGCTATGAAC
Mouse AurkB Reverse	GTTGCCAGGGGTTATGTTTGAG
Mouse Birc5 Forward	AGAACAAAATTGCAAAGGAGACCA
Mouse Birc5 Reverse	GGCATGTCACTCAGGTCCAA
Mouse P53 Forward	CTCTCCCCCGCAAAAGAAAAA
Mouse P53 Reverse	CGGAACATCTCGAAGCGTTTA
Muse Cdkn1a Forward	CGGTGTCAGAGTCTAGGGGA

Mouse Cdkn1a Reverse	ATCACCAGGATTGGACATGG
Mouse Bax Forward	GTGAGCGGCTGCTTGTCT
Mouse Bax Reverse	GGTCCCGAAGTAGGAGAGGA
Mouse Phlda3 Foward	CCGTGGAGTGCGTAGAGAG
Mouse Phlda3 Reverse	TCTGGATGGCCTGTTGATTCT
Mouse Pten Forward	TGGATTCGACTTAGACTTGACCT
Mouse Pten Reverse	GCGGTGTCATAATGTCTCTCAG
Mouse Zmat3 Forward	TTCCTTTACCTAATCGGCCTTCA
Mouse Zmat3 Reverse	TTCCTGCCCAAAAGCCTTCTG

Supplemental Table 15. Sequences of primers used for ChIP-qPCR

Name	Sequence
Human INCENP TSS-216 Forward	ATCTACTTCCTCTCTCGGCC
Human INCENP TSS-216 Reverse	CCAATCCTCGGCAAGTTTGT
Mouse Incenp TSS +755 Forward	CAGACTCATCCCCAGCTCTC
Mouse Incenp TSS +755 Reverse	ATTCCTCACTTCTCCCACCC
Mouse Cdca8 TSS +4391 Forward	CGATTGTTGGTTGCTGGAGT
Mouse Cdca8 TSS +4391 Reverse	TCCTCACACTGGCACTCAAG

SUPPLEMENTAL METHODS

Patient samples

BM samples were collected from 31 hematopoietic stem/progenitor cell (HSPC) donors and 165 newly diagnosed MDS patients with *TP53* wild type according to the Declaration of Helsinki. Informed consent was obtained from all subjects, and the procedures related to primary human samples were approved by the Research Ethics Committee of the First Affiliated Hospital, Zhejiang University School of Medicine (Reference number: 2020-458). Genomic DNA was extracted from the BM mononuclear cells. Targeted Panels with 37 genes frequently mutated in MDS were conducted by targeted next-generation sequencing (NGS). These genes included *ASXL1*, *BCOR*, *CBL*, *CEBPA*, *CREBBP*, *CSF3R*, *CUX1*, *DNMT3A*, *EP300*, *ETV6*, *EZH2*, *FLT3*, *GATA1*, *GATA2*, *IDH1*, *IDH2*, *JAK2*, *KIT*, *KMT2D*, *KRAS*, *NPM1*, *NRAS*, *PHF6*, *PTPN11*, *RUNX1*, *SETBP1*, *SF3B1*, *SRSF2*, *TET2*, *TP53*, *U2AF1*, *WT1*, and *ZRSR2*.

Primary MEF cells, Human CD34+ HPSC and cell line culture

Tp53 WT (C57BL/6) mice, Tp53 KO (jax002101, B6.129S2-Trp53tm1Tyj/J, -/-) male mice, and Tp53 KO (jax002101, B6.129S2-Trp53tm1Tyj/J, +/-) female mice were purchased from Jackson Lab. All mice were bred and housed in specific pathogen-free conditions in the animal barrier facility at Zhejiang Academy of Medical Science, China. And the procedures related to animals were approved by Animal Experimental Ethical Inspection of the First Affiliated Hospital, Zhejiang University School of Medicine (Reference number: 2019-432). Primary mouse embryonic fibroblast (MEF) cells were established and cultured using the standard protocol. MEF cells were isolated from E13.5 embryos obtained from heterozygote breeding. Each embryo was genotyped and individually processed for primary culture. Primers for the Tp53 genotype are listed in Supplemental Table 10. The primary MEF cells were cultured in DMEM containing 10% fetal bovine serum (FBS), 1% Penicillin/Streptomycin, 1x Sodium Pyruvate (Gibco, USA), 1x MEM Non-Essential Amino Acids Solution (Gibco, USA) and 1x GlutaMAX Supplement (Gibco, USA). And the cells were passaged when the cell confluency reached about 80%-90%. Primary MEF were used for a maximum of five passages.

Human CD34+ HPSCs was purchased from Saily Biotechnology Co., Ltd. CD34+ HPSCs were seeded onto a six well plate at a density of 0.8-1.0 × 10^6 cells per ml. of SCGM (CellGenix, #20802-0500), supplemented with three recombinant growth factors of human stem cell factor (100 ng/ml, PEPROTECH, #300-07), Flt3 ligand (100 ng/ml, PEPROTECH, #300-19) and thrombopoietin (100 ng/ml, PEPROTECH, #300-18).

MOLM13, HL-60, HEK293T, and Platinum-E (Plat-E) cell lines were ordered from The American Type Culture Collection (ATCC) and authenticated by short tandem repeat (STR) profiling. The culture conditions of MOLM13 were IMDM medium plus 10% FBS and 1% Penicillin/Streptomycin. HL-60 were cultured in 1640 medium plus 10% FBS and 1% Penicillin/Streptomycin. HEK-293T was cultured in DMEM medium plus 10% FBS and 1% Penicillin/Streptomycin. Platinum-E (PlatE) cells were cultured in DMEM containing 10% Heat-inactivated FBS, 1% Penicillin/Streptomycin, 1x GlutaMax, 1 µg/mL Puromycin, and 10 µg/mL Blasticidin S HCl. These drugs were removed before the transfection. The above cells were cultured in a 37°C, 5% CO2 incubator.

Plasmids of ONECUT3-OE, transfection, and transduction

ORF clone of human *ONECUT3* (NM_001080488.2) was originally synthesized in the pcDNA3.1 vector. pRetroX-TetOne system was gifted by Prof. Kosei Ito (Nagasaki University, Nagasaki, Japan). The full-length, truncated mutants and point mutants of *ONECUT3* were amplified with PCR using the listed primers (Supplemental Table 11) and subcloned into Retro-X-TET-ON system using the BamHI and EcoRI restriction enzyme. Retroviruses were generated by calcium phosphate transient transfection of the retroviral plasmids into PlatE cells. The supernatant was harvested at 48 hours and 72 hours and filtrated with a 0.45 um filter. MEF cells, MOLM13 and HL-60 were plated in a 6-well plate one day before the transduction. On the day of transduction, a fresh medium and retroviral supernatant was added. Polybrene was used at the final concentration of 4 µg/ml. Puromycin (0.5-1 µg/ml) was used for selection to generate stable cell lines and ONECUT3 expression was induced by the doxycycline (100 ng/ml).

Single-stranded oligodeoxynucleotide (ssODN)-mediated ONECUT3 expression in human CD34+ hematopoietic stem/progenitor cells (HSPCs)

To integrate *ONECUT3* targeting at CCR5 locus (1), we constructed the enhanced GFP (EGFP) linked to ONECUT3 expression cassettes driven by PGK promoter and terminated by an SV40 polyA (pA). The EGFP-ONECUT3 cassette was flanked by sequence homologous arms to *CCR5*, at which the single guide RNA (sgRNA, 5′-GCCCAGTGGGACTTTGGAAAT-3′) targeted. Nucleotide sequence of the PGK-EFP-ONECUT3-pA with *CCR5* homologous arms was annealed as ssODN by GeneScript Inc. Sequences and maps of the relevant parts were available in Supplemental Figure 2A.

To achieve efficient genome editing in human CD34+ HPSCs (Saily Biotechnology Co., Ltd.), the optimizing MaxCyte electroporation condition was used to introducing CRISPR/Cas9 system. Two days before electroporation, CD34+ HPSCs were seeded onto a six well plate at a density of 0.8-1.0 × 10^6 cells per ml. of SCGM (CellGenix, #20802-0500), supplemented with three recombinant growth factors of human stem cell factor (100 ng/ml, PEPROTECH, #300-07), Flt3 ligand (100 ng/ml, PEPROTECH, #300-19) and thrombopoietin (100 ng/ml, PEPROTECH, #300-18). On the day of electroporation, CD34+ HPSCs were washed with MaxCyte buffer, and resuspended in MaxCyte buffer at a density of 5 × 10^7 cells/mL. Then 10 μg *Cas9* mRNA (Thermo Fisher, #A29378) and 16 μg *CCR5* sgRNA were incubated for 5 min at room temperature, and then 2.5 × 10^6 cells with 50 μL MaxCyte buffer and 8 μg ssODN were added and mixed gently before transfer to an OC-100 cuvette (MaxCyte, #GOC-1). Immediately after electroporation, the OC-100 cuvettes with HSPCs mixture were incubated at 37°C for 20 mins in a humidified incubator to allow for cell membrane recovery. Finally, cells were added to a six well plate containing 2.5 mL SCGM with three recombinant growth factors as below. Semi-confluent cells (3–4 days after transfection) were harvested for genomic DNA extraction and/or FACS analysis. To detect ONECUT3-targeted integration in *CCR5* locus, genomic DNA of 40–200 ng was analyzed by PCR using primers indicated in Supplemental Table 12. PCR amplicons were resolved on 0.8% agarose gel and visualized by ethidium bromide staining.

The silencing and overexpression of Incenp

Transient knockdown of *Incenp* was performed using siRNAs targeting mouse Incenp or nontargeting siRNA (Supplemental Table 13), delivered by RFect siRNA Transfection Reagent (BAIDAI, Cat#11013). ORF clone of Mus musculus Incenp (NM_016692.4) was originally synthesized in the pMSCV-IRES-GFP II (pMIG II) vector and subcloned into Retro-X-TET-ON system using the BamHI and EcoRI restriction enzyme. The transfection process for packaging retroviruses and subsequent transduction into MEF cells was conducted as above described.

The generation of Anti-ONECUT3 Antibody

Anti-ONECUT3 Antibody was prepared by immunizing rabbits with peptide CMNRWAEEPSTATPG (amino acids 469-482 of human ONECUT3, UniProtKB-O60422) conjugated with keyhole limpet hemocyanin (KLH). The antibody was then purified from rabbit antiserum by the affinity chromatography columns. The procedures of immunization and purification were conducted in Huabio Inc. (Hangzhou, China). In addition, we confirmed the specificity of the antibody by the blocking peptide competition assay and knock-down experiment. The described peptide sequence for generating rabbit anti-ONECUT3 antibody is under China patent application and PCT application (application No. 202111542061.7).

Protein purification of ONECUT3

The procedures of the protein purification of ONECUT3 were conducted in Huabio Inc. (Hangzhou, China). The codon-optimized sequence of the human *ONECUT3* gene (NM_001080488.2) was synthesized and inserted into the pET-28a (+) vector for expression in Rosetta (DE3) cells. To obtain inclusion body proteins, the bacterial strain was initially inoculated in LB medium and grown at 37°C and 220 rpm until the optical density at 600 nm (OD600) reached 0.4-0.6. Subsequently, 1.0 mM IPTG was added to induce the expression of the fusion protein, and the cultivation was continued at 37℃ and 220 rpm for 4 hours. The collected bacteria were subjected to centrifugation, followed by resuspension of the resulting pellet in 1xPBS. The cells were then disrupted through sonication, and another round of centrifugation at 12000 rpm for 5 minutes was performed to separate the supernatant containing soluble proteins

from the pellet containing inclusion bodies. Each fraction was sampled and subsequently analyzed using SDS-PAGE. The inclusion body proteins were purified using Ni-Smart affinity chromatography, employing the following steps: the column was washed with deionized water, and then equilibrated with denaturing equilibration buffer (20 mM PB, 8M Urea, 0.5 M NaCl, pH 7.4). The sample was loaded twice onto a column and collected using a flow-through method. Subsequently, the column was washed three times with deionized water, followed by 0.5M NaOH, and then deionized water again. Finally, the column was equilibrated with 20% ethanol for storage purposes. The loading samples were obtained from the wash fraction and elution fraction after equilibration, with 20 μl reserved for SDS-PAGE analysis. A suitable elution fluid was selected for gradient dialysis, and the target protein was concentrated using an ultrafiltration tube. Ultimately, the protein was preserved in a solution of 1xPBS, 10% Glycerol, pH 7.4.

Surface Plasmon Resonance (SPR)

Surface Plasmon Resonance (SPR) experiments were conducted using a BIAcore T200 (Cytiva) instrument by Target Pharmaceutical (Shanghai) co., Ltd. The CM5 chip (Cytiva) was employed, and a solution containing 50 mM N-hydroxysuccinimide (NHS) and 200 mM 1-ethyl-3-(3-dimethylaminopropyl) carbodiimide (EDC) was introduced onto the sensor surface for a duration of 7 minutes to activate it. Subsequently, the target purified protein ONECUT3 was diluted to a concentration of 20 μg/mL in 10 mM Acetate pH 5.5, with a flow rate of 10 μL/min and a duration of 420 s and immobilized on the CM5 chip surface. Finally, the surface was blocked using 1 M ethanolamine (pH 8.5).

The preliminary determination and manual evaluation of the binding characteristics between the target protein ONECUT3 and the small molecule compound C5484617 were conducted. The highest analytical concentration for the compound was determined to be 12.5 μM. Subsequently, the compound was diluted in a 2-fold gradient, resulting in 8 analytical concentrations ranging from 0 μM to 12.5 μM. The sample analysis was performed with a flow rate of 30 μL/min, a binding time of 120 s, and a dissociation time of 240 s. The experiment was carried out employing multiple cycles, wherein the response signal was graphed against analysis time. The

acquired data were subjected to fitting using the BIAcore T200 analysis software, employing a 1:1 Langmuir binding model to ascertain kinetic constants such as the association rate constant, dissociation rate constant, and binding and dissociation equilibrium constants.

Drug Affinity Responsive Target Stability (DARTS) for target identification

The target identification of the small-molecule C5484617 were conducted using Drug Affinity Responsive Target Stability by Target Pharmaceutical (Shanghai) co., Ltd. The protocol is as reference (2) . 293T cells were lysed in M-PER buffer with the addition of both protease inhibitors and phosphatase inhibitors. After chilled TNC buffer (50 mM Tris-HCl pH 8.0, 50 mM NaCl, 10 mM CaCl2) was added to the protein lysate, the protein concentration of the lysate was measured by the BCA Protein Assay kit (Pierce, 23227). The protein lysate was then incubated for 1 hours at room temperature with either vehicle control (DMSO) or 100 μM compound C5484617; materials were shaken at 600 rpm in an Eppendorf Thermomixer. Pronase digestions (1:200), which were performed for 30 min at room temperature, were stopped by adding SDS loading buffer and immediately heating at 70 °C for 10 min. Samples were both subjected to SDS–PAGE.

For the decolorization of the gel strip, the reduction and alkylation process involved the use of DTT and Iodoacetimide. Subsequently, a trypsin enzyme solution was introduced, and the mixture was kept at a temperature of 4°C for a duration of 60 minutes, followed by overnight digestion at 37°C. To extract the digested peptides, a blend of formic acid and acetonitrile was prepared and subjected to ultrasonic treatment at 37°C. The resulting mixture of peptides was then dried and reconstituted in a 0.1% formic acid (FA) solution. After vacuum drying, the sample was dissolved in FA once again, and an equal volume of the sample was extracted for mass spectrometry analysis using the Q-Exactive HF system (Thermo Scientific, USA). The sample was subjected to separation using the EASY-nLC 1200 system (Thermo Scientific, USA), employing a C18 analytical column (1.9μm particle size, 75 μm×15 cm) at a flow rate of 300 nL/min. Subsequently, tandem mass spectrometry detection was performed in Data Dependent Acquisition (DDA) mode.

Protein extraction and immunoblotting

The cell pellet was resuspended in SDS lysis buffer (50 mM Tris-HCl pH 6.8, 2% SDS, 10% glycerol, 2% β-mercaptoethanol and 0.02 % bromophenol blue) with Protease Inhibitor (Abcam) PhosSTOP plus Phosphatase Inhibitor Cocktail (Roche) and followed by sonication. Samples were heated at 95°C for 10min and loaded to SDS-PAGE gel. Followed by electrophoresis, transfer-membrane, and blocking, the primary antibodies were incubated at 4 °C overnight. The following primary antibodies (listed in Supplemental Table 8) were used in this study: anti-FLAG (Sigma, F3165), anti-INCENP (Abcam, ab12183), anti-Aurora B (Abcam, ab2254), anti-Borealin (Santa Cruz, sc-376635) and anti-Survivin (Huabio, ET1602-43). In addition, diluted horseradish peroxidase (HRP)-conjugated secondary antibodies were incubated for one hour at room temperature, and SuperSignal West Dura or Femto Chemiluminescent Substrate (Thermo Fisher) were used for ECL detection. The membranes were imaged and visualized in ChemiDoc MP Imaging System with ImageLab 5.0 software (Bio-Rad). Protein bands were quantified by measuring the peak area via ImageJ (NIH).

Analysis of cell viability, colony formation assay, apoptosis, and necrosis

0.3 M cells were plated in each well of 6-well plates for three replicates, and the overexpression of ONECUT3 was induced using 100 ng/μl Doxycycline for 0–8 days. The cell number was counted every two days, and the culture medium was refreshed simultaneously. For drug treatment, ten thousand cells were plated in 96-well plates in the presence of the chemotherapeutic agents (listed in Supplemental Table 9). The plates were incubated at 37 °C for 48 hours. After the drug treatment, cell viability was measured utilizing the CellTiter-Lumi™ Plus Luminescent Cell Viability Assay Kit (Beyotime, Shanghai, China) according to the protocol. One thousand MOLM13 cells, after two days of the induction of ONECUT3 overexpression, were plated in triplicate into 1 ml methylcellulose-based medium (Stem Cell Technologies, MethoCult GF M3434). After 14 days, the colonies were scored, and images of the representing colonies were captured by an ECLIPSE E400 microscope (Nikon, Japan). Apoptosis and necrosis were conducted by Annexin V-APC/7-AAD staining Kit (BD, 550474). Flow cytometry was performed

on a BD FACS-Canto II Flow Cytometer (BD) and analyzed with FlowJo 10.3 (TreeStar).

Wright-Giemsa staining

MEF cells were washed with PBS and harvested by trypsin. Place 0.3 M cells (in 100 µl medium per slide) in Cytospin 4 Centrifuge (Thermo Scientific, USA), centrifuge them at 500x rpm for 3 minutes, and dry them at room temperature for 20-30 minutes. The slides were then stained by Camco Stain Pak (Camco, USA) according to the manual. Briefly, fix them in Fixative Solution for 10 seconds; then dye in Solution I and II for 30 seconds and 20 seconds, respectively. Cell morphology was photographed in a 100x oil len by an ECLIPSE E400 microscope (Nikon, Japan).

Total RNA extraction, reverse transcription, and qPCR assay

Total RNA was extracted using Trizol reagent (Invitrogen) and the RNeasy Mini Kit (Qiagen). Then 1 µg of total RNA was reverse transcribed into cDNA using the SuperScript IV First-Strand Systhesis System (Invitrogen). Primers for qPCR are listed in Supplemental Table 14. Real-time PCR was conducted using the TB Green Premix Ex Taq (Takara-Bio) on the Applied Biosystems 7500 Fast Real-Time PCR System (Applied Biosystems). The relative mRNA expression level of each gene was calculated as 2-△△Ct to the internal reference (ACTIN).

RNA sequencing (RNA-seq)

RNA-seq was conducted by the Genomics, Epigenomics and Sequencing Core of the University of Cincinnati (Cincinnati, Ohio). NEBNext Ultra II Directional RNA Library Prep Kit was used for library preparation, and adapter trimmed reads were generated in fastq format. Demultiplexing is performed under the Illumina BaseSpace default setting. Adapter: AGATCGGAAGAGCACACGTC; AdapterRead2: AGATCGGAAGAGCGTCGTGT. RNA-seq was performed using the NextSeq 550 platform (Illumina, San Diego, CA). General bioinformatic analysis was performed via SEQUENCE HUB app RNA-Seq Alignment v2.0.2 followed by RNA-Seq Differential Expression version 1.0.1. The analysis used STAR for alignment and Salmon for quantification (Transcripts Per Million, TPM), followed by DESeq2 to identify differentially expressed genes. Afterward, the significantly regulated biological processes were identified by

GO term analysis and Gene-set enrichment analysis (GSEA).

Chromatin Immunoprecipitation sequencing (ChIP-seq) and ChIP-qPCR Assay

MEF cells were crosslinked with 1% formaldehyde at room temperature for 10 minutes. 1.5 M Glycine solution was used to terminate the fixation reaction. After washing with the iced PBS and homogenizing, cells were then lysed with SDS cell-lysis buffer (50 mM Tris pH 8.0, 1 mM EDTA pH 8.0, 0.1% wt/vol SDS, and 1 mM PMSF) for 10 min on ice, and the chromatin was sonicated to 100–500bp in a Bioruptor (Diagenode). The size of the sheared chromatin was verified by agarose gel electrophoresis after reverse crosslinking.

For immunoprecipitation, Dynabeads Protein G (Thermo, 10003D) was pre-washed was 0.5% BSA and conjugated to 5 μg of ChIP antibody or normal IgG at 4 °C for 4-6h. After washing with the RIPA/150mM NaCl washing buffer (50 mM Tris pH 8.0, 150 mM NaCl, 1 mM EDTA pH 8.0, 1% wt/vol sodium deoxycholate, 1% vol/vol Triton X-100, 0.1% wt/vol SDS and 1 mM PMSF), the conjugated beads were added to the chromatin slurry and incubated at 4 °C for 12 hours. Afterward, beads were washed twice with RIPA/150mM NaCl washing buffer, and twice with RIPA/500mM NaCl washing buffer (50 mM Tris pH 8.0, 500 mM NaCl, 1 mM EDTA pH 8.0, 1% wt/vol sodium deoxycholate, 1% vol/vol Triton X-100, 0.1% wt/vol SDS and 1 mM PMSF), four times LiCl washing buffer (10 mM Tris pH 8.0, 250 mM LiCl, 1 mM EDTA pH 8.0, 0.5% vol/vol NP-40 and 0.5% vol/vol Sodium Deoxycholate) and twice with TE buffer (10 mM Tris pH 8.0, 300 mM NaCl and 0.5 mM EDTA pH 8.0). The precipitated beads were diluted with ChIP elution buffer (10 mM Tris pH 8.0, 250 mM LiCl, 1 mM EDTA pH 8.0, 0.5% vol/vol NP-40, and 0.5% wt/vol SDS). One-fourth of the solution with beads was added with SDS protein lysis buffer for WB to verify the specificity of ChIP. The rest of the ChIP'd beads and the input were reverse crosslinking with 5 M NaCl solution at 65 °C overnight.

The procedure for DNA recovery from immunoprecipitation is as follows. First, the above reversed crosslinking solution was mixed with 2x SDS lysis buffer (200 mM Tris pH 8.0, 1 M NaCl, 100mM EDTA pH 8.0, and 0.5% wt/vol SDS) with Proteinase K at 200x rpm for 15 minutes and

followed by RNase A at 37 °C for 30 minutes. Next, 3 M NaAc (pH 5.2) was used to remove the protein. The clean supernatant was then mixed with Isopropanol and Glycogen to precipitate the DNA. After being washed twice with pre-cooled 75% ethanol, DNA pellets were dried for 10 minutes, dissolved in 10 mM Tris-HCl (pH 8.0), and then measured by the Qubit dsDNA HS Assay Kit (Thermo Fisher) on the Qubit instrument (ThermoFisher Scientific). Finally, the ChIP'ed DNA proceeded for ChIP-seq or ChIP qPCR.

NEBNext Ultra II DNA Library Prep Kit for Illumina (NEB, E7645) was used for library preparation ChIP-seq was performed using the HiSeq2500 platform with paired-end 100 bases (Illumina) at the DNA sequencing core facility of the Cincinnati Children's Hospital (Cincinnati, Ohio). All sequencing data were mapped to mouse genome assembly GRCm38. Reference genome and gene model annotation files were downloaded from the genome website directly. Paired-end clean reads were aligned to the reference genome using Bowtie v2.2.6. After mapping reads to the reference genome, we used the MACS2 version 2.1.0 (model-based analysis of ChIP-seq) peak-finding algorithm to identify regions of IP enrichment over the background. A p-value threshold of enrichment of 0.01 was used for all data sets. Then, the distribution of chromosome distribution, peak width, fold enrichment, significant level, and peak summit number per peak were all displayed. For all ChIP-seq data sets, normalized bigWig files were generated by deep tools (version 3.3.2 https://deeptools.readthedocs.io/en/develop/), which were subsequently visualized using IGV version 2.8.13.

ChIP-qPCR was performed similarly to the ChIP-seq experiments before the library preparation. Primers for ChIP-qPCR are listed in Supplemental Table 15. Real-time PCR was conducted using the TB Green Premix Ex Taq (Takara-Bio) on the Applied Biosystems 7500 Fast Real-Time PCR System (Applied Biosystems). '% Input' was used to show the enrichment.

Flow Cytometry and FACS

The BM mononuclear pellets were incubated with Zombie Aqua (Biolegend, San Diego, CA) and FcR block (BD) for 10 min at 4 °C. For surface marker staining, cells were incubated with

fluorescence-conjugated antibodies (FITC-lin, PB-CD38, and APC-CD34) for 30-60 min at 4 °C. The lineage cocktail for human cells included antibodies against CD2, CD3, CD7, CD10, CD11b, CD14, CD19, and CD235a. The cells were fixed and permeabilized by eBioscience™ FOXP3/Transcription Factor Staining Buffer set (Thermo Fisher Scientific) for intracellular staining of transcriptional factors according to the manufacturer's protocol. Then, the cells were stained with ONECUT3-PE antibody or isotype IgG-PE for 30 min. Flow cytometry was performed with a Cytoflex cytometer (Beckman Coulter, Brea, CA) and analyzed with FlowJo 10.3 (TreeStar).

Quantitation of mitotic cells

Cells were processed for flow cytometry to quantitate the number of mitotic cells as described previously (3). Briefly, cells were fixed in pre-cold 90% methanol/10% PBS for 15min at -20°C. The PBS-washed cells were incubated with an antibody cocktail for 1 hour at 37°C. The formula of antibody cocktail: primary antibody (phospho-(Ser10)-histone H3, Abcam, ab5176, 1:100) in the buffer (PBS + 3% w/v bovine serum albumin + 0.05% Tween 20 + 0.04% Sodium Azide). Then cells were washed with PBS and incubated with the FITC-conjugated secondary antibody for 30 min at 37°C. After centrifugation, the cells were added with DNA labeling solution (0.5 μg/ml propidium iodide with RNAse A) and incubated for 15 minutes at 37°C. Flow cytometry was performed on a BD FACS-Canto II Flow Cytometer (BD) and analyzed with FlowJo 10.3 (TreeStar).

Chromosome spreads

Cells were treated with 150 ng/ml (final concentration) colcemid for 2 hours at 37°C. Then the cells were digestion by the trypsin and swollen in a pre-warmed hypotonic solution (0.075 M KCl) for 20 min at 37°C. Next, cells were centrifuged at 1000 G for 7 min, followed by two rounds of fixation in ice-cold Carnoy’s fixative for 10 min at room temperature. Cells were dropped on glass slides, stained in DAPI or Giemsa, dried, and scanned.

Immunohistochemistry (IHC)

The formalin-fixed and paraffin-embedded BM tissue from MDS patients were cut into 3 μm thin sections. These sections were rehydrated and underwent antigen retrieval, followed by antigen blocking. They were subsequently incubated with primary anti-ONECUT3 antibody (homemade) and Aurora B (Abcam, ab2254) at 4 °C overnight and incubated with HRP-conjugated antibody against rabbit IgG with DAB as the chromogen. The whole slides were digitally scanned using Digital Pathology 5 Slides scanner (KFBIO, Ningbo, China) under 40x objective magnification. The cells were automatically separated, detected, and quantified by the QuPath v0.2.3 image analysis software (Queen's University, Northern Ireland) under the supervision of a pathologist (4). The ONECUT3 and Aurora B expression level of immunohistochemistry staining was evaluated by the staining intensity and positive cell percentage from the total slides.

SUPPLEMENTAL REFERENCES:

1. Lombardo A, et al. Site-specific integration and tailoring of cassette design for sustainable gene transfer. *Nat Methods.* 2011;8(10):861-869.
2. Pai MY, et al. Drug affinity responsive target stability (DARTS) for small-molecule target identification. *Methods Mol Biol.* 2015;1263:287-298.
3. Andreassen PR, et al. Neither p21WAF1 nor 14-3-3sigma prevents G2 progression to mitotic catastrophe in human colon carcinoma cells after DNA damage, but p21WAF1 induces stable G1 arrest in resulting tetraploid cells. *Cancer Res.* 2001;61(20):7660-7668.
4. Bankhead P, et al. QuPath: Open source software for digital pathology image analysis. *Sci Rep.* 2017;7(1):16878.
